# Blood and lymphatic vascular remodeling in intervertebral disc degeneration

**DOI:** 10.3389/fimmu.2026.1862921

**Published:** 2026-08-12

**Authors:** Fei Huang, Maoqiang Lin, Qiang Zhang, Shishun Yan, Rui Li, Shaolong Li, Yunbin Jia, Qiao Zhao, Pengjie Song, Haiyu Zhou

**Affiliations:** 1 Department of Orthopedics, Lanzhou University Second Hospital, Lanzhou, Gansu, China; 2 Key Laboratory of Orthopedics Disease of Gansu Province, Lanzhou University Second Hospital, Lanzhou, Gansu, China; 3 Department of Spinal Surgery, The First People’s Hospital of Xianyang, Xianyang, China

**Keywords:** blood vessels, discogenic pain, intervertebral disc degeneration, lymphatic vessels, vascular system, VEGF

## Abstract

A defining histological feature of the intervertebral disc (IVD) in healthy adults is its avascular and alymphatic status, which is closely related to its mode of nutrient supply and local immune homeostasis. Following the onset of intervertebral disc degeneration (IDD), blood vessels invade the interior of the degenerated IVD under the combined effects of anatomical barrier disruption, inflammatory responses, and aberrant activation of growth factor regulatory networks, thereby accelerating the degenerative process and being closely associated with discogenic pain. However, as another major component of the vascular system, there is still no consensus regarding the presence of lymphatic vessels in the adult IVD, their spatial distribution, and whether they serve functional drainage and immunoregulatory roles. In addition, differences among studies in species background, tissue sampling range, degeneration stage, and detection strategies have further increased the complexity of mechanistic interpretation. Against this background, this review first outlines the vascular anatomical and physiological basis of the normal IVD, summarizes vascular-related pathological changes and key regulatory networks during IDD, and synthesizes the principal findings, major controversies, and methodological limitations of lymphatic research. On this basis, it further discusses the potential links between remodeling of the vascular system, including both blood vessels and lymphatic vessels, and pain generation as well as degenerative outcomes, and outlines potential intervention strategies targeting vasculature-related pathways, with the aim of informing subsequent mechanistic studies and translational therapeutic development.

## 1 Introduction

Low back pain (LBP) has long ranked among the leading causes of disability in global burden of disease assessments and shows an increasing trend with population aging and cumulative occupational exposure (1–3). Among the multifactorial etiological spectrum, intervertebral disc degeneration (IDD) is considered an important structural basis of LBP (4). However, the key driving mechanisms of IDD have not yet been fully elucidated, and current treatments still mainly focus on symptom relief or end-stage surgery, with a relative lack of effective mechanism-based interventional strategies targeting the degenerative process (5–7). Therefore, identifying the core pathological events that regulate IDD progression and are amenable to translational therapy is a scientific issue that urgently needs to be addressed.

The healthy adult intervertebral disc (IVD) is generally considered the largest avascular and alymphatic tissue in the human body, and its nutrient supply and metabolic waste clearance mainly depend on diffusional exchange through the cartilage endplates (CEPs) and the outer annulus fibrosus (AF) (8–10). This exchange pattern shapes the unique hypoxic and acidic metabolic microenvironment of the IVD and structurally establishes a relatively “immune-privileged” state (9, 11, 12). Once the avascular and alymphatic structure is disrupted, it may trigger microenvironmental imbalance, inflammatory responses, and tissue remodeling, thereby promoting degenerative progression (11, 13).

A large body of evidence indicates that pathological angiogenesis is an important characteristic event in IDD (14–17). During the degenerative process, newly formed blood vessels can extend into the nucleus pulposus (NP) and the inner AF, accompanied by amplification of inflammation, matrix degradation, and local metabolic alterations (18). Meanwhile, nerve fibers often enter in coordination with blood vessels, forming “neurovascular complexes” and providing a potential anatomical basis for discogenic pain (16). However, the existing evidence still leaves room for interpretation regarding whether vascular and neural ingrowth is a driving factor in IDD or a reactive consequence of AF fissures, endplate injury, and the inflammatory microenvironment.

As another key component of the vascular and lymphatic systems, the existence, spatial distribution, and functionality of lymphatic structures in the IVD still lack a unified conclusion. Given the known functions of the lymphatic system in fluid drainage, inflammatory regulation, and tissue repair (19), exploring its role in microenvironmental remodeling during IDD may contribute to a more comprehensive understanding of the mechanisms underlying degeneration. On this basis, this article focuses on the structural and functional changes in the vascular and lymphatic systems of the IVD during the degenerative process, reviews the molecular regulation of pathological angiogenesis and its role in IDD, summarizes the major findings and controversies in lymphatic-related research, and discusses potential intervention strategies targeting the vascular and lymphatic systems, with the aim of providing a reference for mechanism-oriented research design and therapeutic translation.

## 2 Vascularity and nutrient supply of the normal intervertebral disc

**Figure 1:**
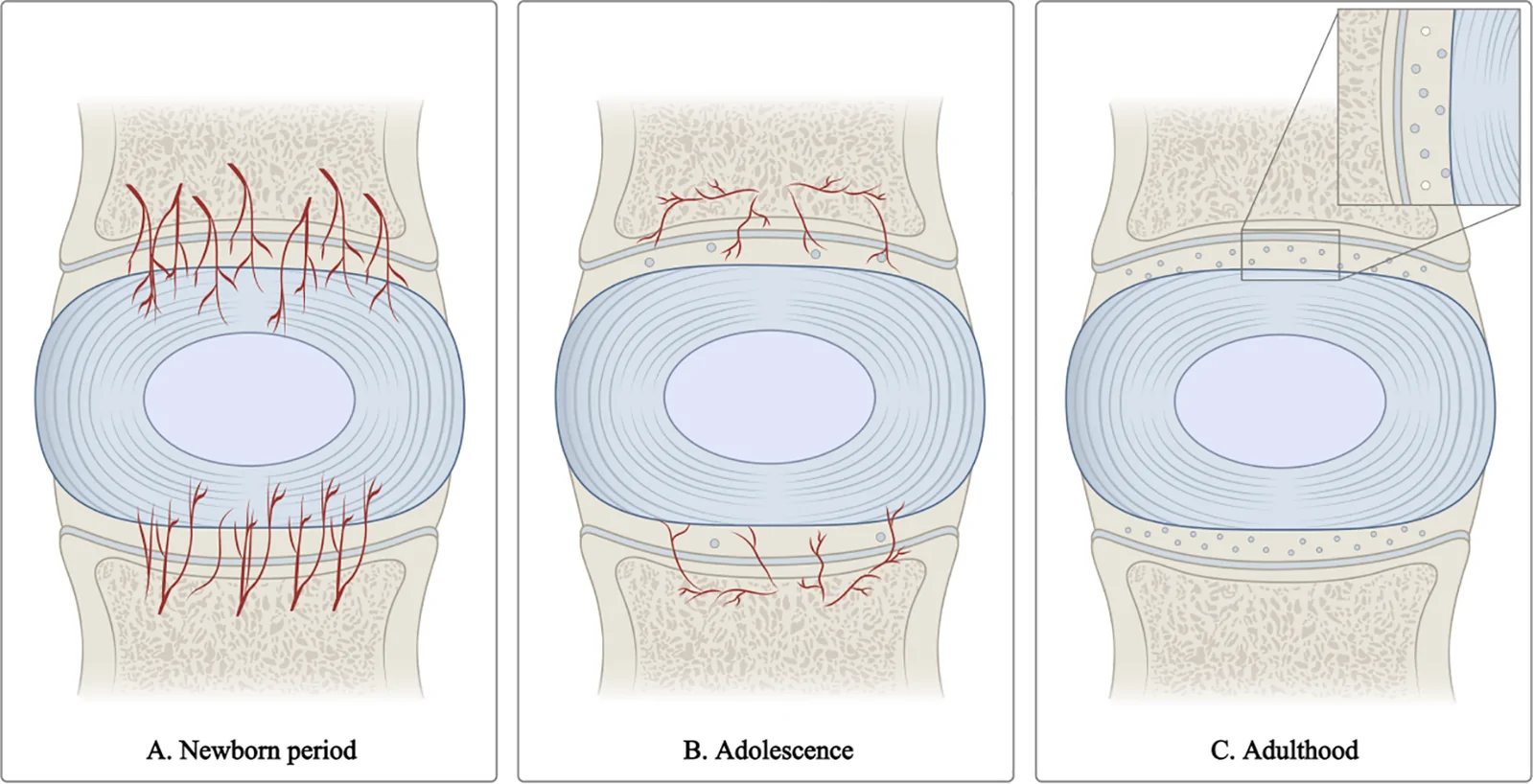
Vascular changes in the development of the intervertebral disc. **(A)** In the neonatal stage, the intervertebral disc is relatively well vascularized, with blood vessels distributed within the disc and adjacent to the cartilaginous endplate. **(B)** After adolescence, vascularity within the disc progressively decreases and regresses, and the blood supply becomes mainly restricted to the endplate region. **(C)** In adulthood, the mature intervertebral disc generally lacks direct vascularization, and tissue metabolism depends primarily on nutrient diffusion mediated by the cartilaginous endplate. The inset in the upper right illustrates the microporous architecture remaining after degeneration of the cartilaginous endplate.

The IVD consists of the central NP, the peripheral AF, and the superior and inferior CEPs, and its blood supply is mainly derived from segmental arteries and intervertebral arteries arising from the aorta (20). During development, the vascular distribution within the IVD exhibits marked stage specificity (**Figure 1**): abundant vascularization is present during early embryonic development, the fetal period, and the neonatal period; after birth, these vessels gradually regress, with the vasculature in the NP and AF disappearing at approximately 4 years of age, whereas the vasculature in the CEPs gradually closes during the first two decades of life; in adulthood, the NP, inner AF, and CEPs remain avascular, but micropores left by vascular regression within the CEPs are retained and become the principal pathways for IVD metabolism (21–24). Therefore, although a small number of microvessels are retained in the outermost AF (within an approximately 1–2 mm range) in some individuals, the avascular state is still regarded as a typical feature of the adult IVD.

The nutrient supply of the IVD is mainly derived from the microvascular beds in the marrow cavities of the bony endplates, and substance exchange depends on the permeability of the CEPs, whereas the contribution from microvessels in the outermost AF is relatively limited (25). Because there is a distance of 6–8 mm between the cells in the central region and the nearest perfusable microvessels, diffusional efficiency is constrained; therefore, the interior of the IVD maintains a chronically hypoxic and relatively acidic microenvironment and exhibits a glycolysis-dominant metabolic pattern (26). A three-dimensional model of fluid distribution in the IVD constructed on the basis of MRI showed that the fluid content in cephalad IVDs was higher than that in caudal segments (27), suggesting that differences in microvascular nourishment or material transport efficiency may exist among different segments. Such differences may be associated with the higher incidence of degeneration in caudal segments, although their causal relationship still requires further verification.

Maintenance of the homeostasis that “restricts vascular entry” in the adult IVD involves multiple mechanisms, including matrix barriers (28), anti-angiogenic signaling (29), and cell–endothelial interactions (30). The extracellular matrix (ECM) of NP cells is mainly composed of aggrecan and type II collagen: aggrecan maintains the avascular state of the NP through anti-angiogenic effects, whereas type II collagen primarily maintains IVD morphology and resists tensile forces (31–33). The ECM of the CEPs not only regulates substance transport but also resists microvascular neogenesis by inhibiting vascular endothelial growth factor (VEGF) (34, 35). In addition to serving as a physical barrier, the AF also secretes exosomes that inhibit vascular ingrowth (29).

In addition to ECM- and paracrine-mediated regulation, the Fas apoptotic pathway also plays a key role in preventing neovascularization. Sun et al. (30) established a coculture system of NP cells and vascular endothelial cells and found that normal NP cells had a stronger ability to induce apoptosis in vascular endothelial cells than degenerative NP cells. FasL can mediate the activation of downstream FADD and caspase-3, thereby inducing apoptosis in vascular endothelial cells and preventing vascular regeneration in the IVD.

## 3 Mechanisms of angiogenesis in intervertebral disc degeneration

**Figure 2:**
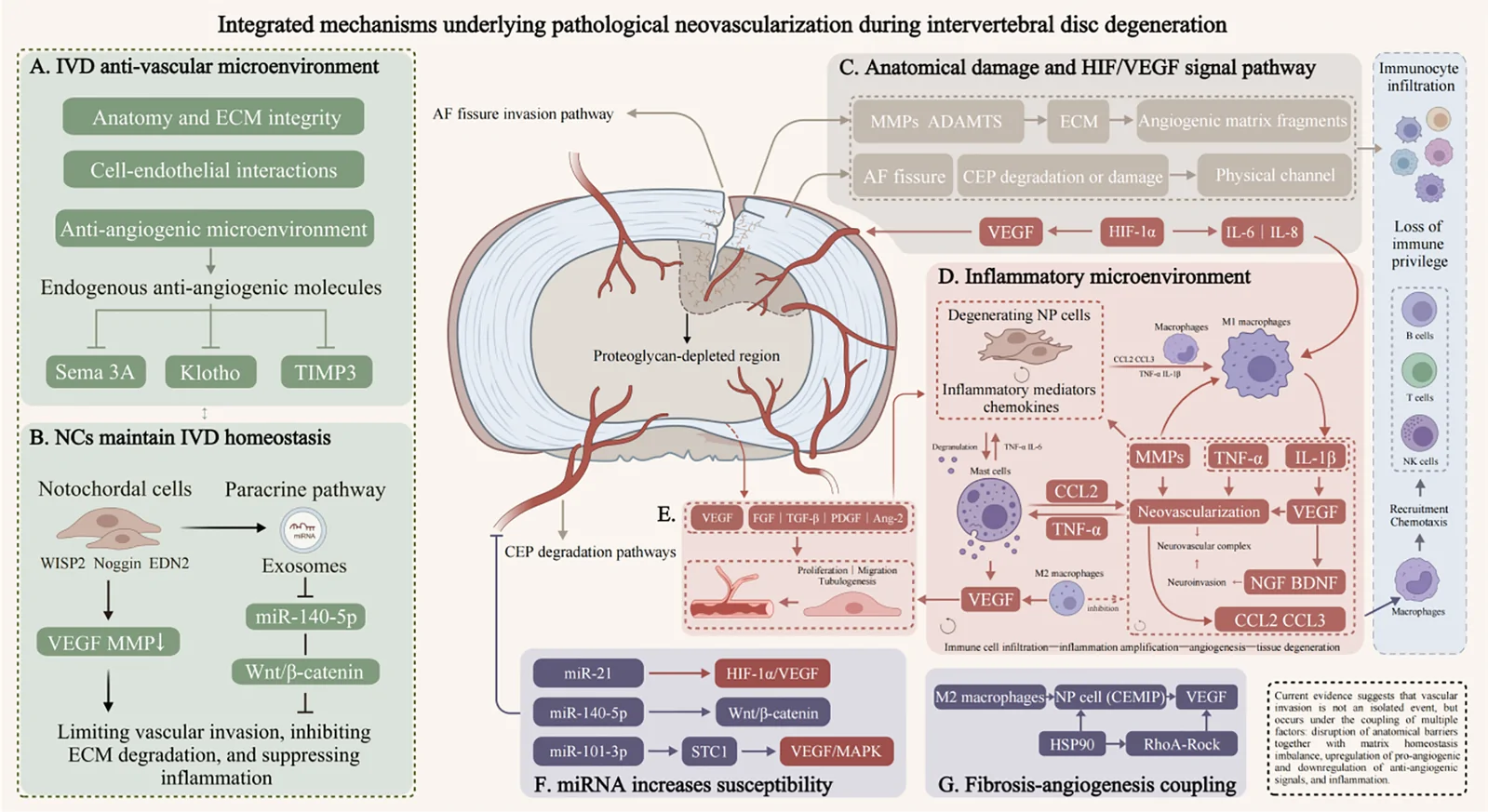
Integrated mechanisms underlying pathological neovascularization during intervertebral disc degeneration. **(A)** The healthy intervertebral disc (IVD) maintains an anti-vascular microenvironment through intact anatomical barriers, extracellular matrix (ECM) integrity, cell–endothelial interactions, and endogenous anti-angiogenic molecules, including semaphorin 3A (Sema3A), Klotho, and TIMP3. **(B)** Notochordal cells (NCs) preserve IVD homeostasis via paracrine signaling and exosome-mediated regulation, thereby limiting vascular invasion, extracellular matrix degradation, and inflammation. **(C)** During degeneration, annulus fibrosus (AF) fissures, cartilaginous endplate (CEP) damage, and matrix degradation mediated by MMPs and ADAMTS generate physical channels and angiogenic matrix fragments; meanwhile, HIF-1α/VEGF signaling and inflammatory cytokines further promote neovascularization. **(D)** The inflammatory microenvironment, involving degenerative nucleus pulposus (NP) cells, macrophage polarization, and mediators such as CCL2, TNF-α, IL-1β, and MMPs, amplifies vascular invasion, immune cell recruitment, and neural ingrowth. **(E)** CEP degeneration promotes angiogenic activation through VEGF, FGF, TGF-β, PDGF, and Ang-2, thereby enhancing endothelial proliferation, migration, and tubulogenesis. **(F)** Dysregulated microRNAs increase susceptibility to neovascularization by modulating multiple signaling pathways, including HIF-1α/VEGF, Wnt/β-catenin, STC1, and VEGF/MAPK. **(G)** Fibrosis–angiogenesis coupling further drives vascular ingrowth through interactions among M2 macrophages, NP cells, CEMIP, VEGF, HSP90, and RhoA/ROCK signaling.

During the progression of IDD, the appearance of neovessels in the originally avascular NP and inner AF is regarded as one of the important pathological features (14–17). Existing evidence indicates that vascular invasion is not an isolated event but occurs through the coupled effects of multiple factors, including disruption of anatomical barriers and imbalance of matrix homeostasis (36, 37), upregulation of pro-angiogenic signals (38), downregulation of anti-angiogenic signals (39), and inflammatory responses (40)(**Figure 2**).

### 3.1 Disruption of anatomical barriers and imbalance of matrix homeostasis

During the progression of IDD, the AF may develop radial or concentric fissures, and the CEPs may also undergo changes such as microfractures, structural disruption, or rupture of the calcified plate (17, 36). Previous studies have found that neovessels are more commonly observed near AF fissures, in regions of proteoglycan depletion, and in granulation-like tissue within the NP, whereas they are less frequent in regions with relatively preserved structure (36, 37). This distribution pattern suggests that structural damage weakens the original anatomical boundaries and barrier function of the IVD, providing potential physical channels for peripheral vessels to extend into regions that are originally relatively avascular.

The fissures formed by structural disruption not only provide channels but also alter the local mechanical and chemical environment (36). Fissured regions are usually accompanied by focal loss of proteoglycans and a decrease in matrix water content, and the local compressive stress is also lower than that in the surrounding normal tissue (36, 37). A low-pressure environment is conducive to maintaining the patency of the microvascular lumen, whereas proteoglycan reduction weakens its inhibitory effects on vascular and nerve growth (31, 41). Meanwhile, the activity of degradative enzymes such as matrix metalloproteinases (MMPs) is increased in degenerative IVDs, leading to ECM degradation (42). Loss of ECM integrity not only weakens its physical barrier function but also releases matrix fragments with pro-angiogenic activity during the degradation process, further amplifying angiogenic signaling (43). After vascular invasion, inflammatory cells and soluble factors can also enter the interior of the IVD, promoting persistent matrix remodeling and inflammatory responses, forming a feedback loop, and accelerating the degenerative process (44).

In addition to the fissure pathway of the AF, CEP degeneration may also constitute another source of vascular entry: when endplate integrity is compromised or related vascular channels undergo abnormal dilation, vessels from the vertebral side may extend inward along the endplate–nucleus pulposus interface (45). Endplate ossification and microcalcification are important changes associated with IDD (46, 47). Studies have suggested that early endplate calcification aggravates local nutritional impairment by narrowing the vascular channels of the bony endplate and reducing diffusion efficiency (47). However, during stages of aggravated degeneration and inflammation, calcified crystals may act as a source of chronic inflammatory stimulation, upregulate the expression of pro-angiogenic factors such as VEGF, and create conditions for endothelial cell migration by altering ECM structure (48–50). Clinicopathological studies have shown that the degree of microcalcification is positively correlated with vascular density and that angiogenesis is more pronounced in non-contained herniated tissue that has breached the posterior longitudinal ligament (49), suggesting that when degenerative tissue crosses the original anatomical boundary and comes into contact with a blood-rich environment, it is more likely to trigger an angiogenic response. Taken together, vascular invasion during the degenerative process does not occur uniformly but exhibits a certain degree of spatial selectivity.

This spatial selectivity also complicates any simple correspondence between the extent of vascular ingrowth and the severity of IVD degeneration. Lama et al. quantified CD31-positive microvessels by confocal microscopy in 40 surgical specimens and found that vascular density increased with higher Pfirrmann grade and greater proteoglycan loss (37). Ozden et al. similarly observed a positive correlation between semi-quantitative CD34 staining and Pfirrmann grade (R = 0.604, P < 0.001) in 32 L4-L5 herniations spanning grades I-IV (51). Rätsep et al. using a histological degeneration score rather than imaging-based grading, likewise found that neovascularization correlated with the degree of tissue degeneration in 50 specimens (49). Munarriz et al. however, assessed 122 surgical patients with Pfirrmann grading and Weiler histological scoring evaluated independently by two observers and detected no significant association between neovascularization and Pfirrmann grade (52). These studies suggest that MRI grading and histological assessment of surgically resected tissue differ in both the scale of evaluation and the scope of tissue sampling: Pfirrmann grading primarily reflects global changes in disc signal, morphology, and height, whereas histological examination captures focal, heterogeneous microvascular reactions within herniated tissue. When vascular ingrowth depends on annular fissures, herniation exposure, or local structural disruption, its extent may not correspond linearly with the imaging grade of the entire disc.

The temporal position of angiogenesis in degeneration remains controversial. Most studies suggest that it mainly occurs during the moderate-to-severe stages of degeneration, whereas some angiographic and cadaveric studies indicate that occlusion of the anastomotic arteries of the posterior longitudinal ligament and neovascularization in the anterolateral intervertebral space may precede overt histological signs of degeneration (14, 49). This finding supports an explanatory framework of “adaptive collateral reconstruction–secondary pathological transformation”: early angiogenesis may arise from a compensatory response to segmental blood supply restriction, but, in the context of persistent inflammation and matrix imbalance, it may transform into a pathological pathway that promotes nerve ingrowth and inflammatory cell infiltration. Therefore, further elucidation of the key molecular basis driving angiogenesis in the above context is of great significance for understanding the pathological mechanisms of angiogenesis in IDD.

### 3.2 Angiogenesis-related growth factors

#### 3.2.1 Pro-angiogenic growth factors

##### 3.2.1.1 Vascular endothelial growth factor

VEGF is a core regulatory factor driving angiogenesis and mainly promotes endothelial cell proliferation, migration, and tube-like structure formation by activating vascular endothelial growth factor receptor-2 (VEGFR-2) on the surface of endothelial cells (53). Within the IVD, VEGF is not merely a pathological molecule; its basal expression is of certain significance for maintaining local cell survival and tissue biological function (15, 54). With the progression of IDD, VEGF levels in the IVD increase, and enhanced VEGF expression is associated with the degree of degeneration (55). Xiao et al. (38) established a mouse model of degeneration and, 12 weeks after successful model induction, harvested the L1–6 spinal segments for immunofluorescence analysis. The results showed that VEGF expression was significantly increased in moderately to severely degenerated IVDs, with particularly prominent positive signals in the NP region. Further studies found that the regions with high VEGF expression showed a certain spatial correspondence with regions exhibiting abnormal matrix component expression and osteochondral-like remodeling, providing supportive evidence for the spatial selectivity of the vascular distribution described above.

In the early stage of degeneration, NP cells gradually undergo phenotypic abnormalities and secrete VEGF, thereby inducing endothelial cell migration; this process is considered one of the potential initiating events of microvascular invasion into the IVD (54, 56). Zhan et al. (35), using an ex vivo IVD culture model, confirmed that VEGF expression levels were significantly correlated with the number of vessels in degenerative IVDs; meanwhile, regulation of VEGF expression in CEP cells could improve the state of vascular sprouting and influence the progression of IDD. A study by Sun et al. (29) showed that degenerative AF cells can secrete exosomes, upregulate the expression of VEGF, TNF-α, IL-6, and MMPs, induce vascular endothelial cell migration, and ultimately promote microvascular ingrowth into the IVD. In addition, upregulation of VEGF in degenerative IVDs can induce the invasion of neomicrovessels into the cartilaginous matrix of the CEPs, whereas degenerative CEP cells can also promote the migration of endothelial cells and osteoclasts to this region by secreting VEGF-A. These processes may ultimately lead to loss of CEP function and structural remodeling as a result of microvascular invasion, accompanied by significantly increased expression of VEGF-A, CD31, CD34, and vascular endothelial cadherin in the CEPs (57).

However, after systematically analyzing human IVD tissues with different grades of degeneration, Simona et al. (15) found that the expression of the classical VEGF pathway remained stable across all stages and did not change significantly with the progression of degeneration. This finding challenges the traditional view that the VEGF pathway drives degenerative angiogenesis. The study further revealed that, in severely degenerated IVDs, alternative signaling pathways associated with aberrant angiogenesis are activated, particularly with significant upregulation of hypoxia-inducible factor-1α (HIF-1α) and the serine protease HTRA1. These findings suggest that pathological angiogenesis in the late stage of IDD may be dominated by these alternative pathways triggered by the hypoxic microenvironment, rather than by the classical VEGF pathway, thereby providing a new direction for understanding the mechanisms of angiogenesis in IDD.

In addition to its angiogenic effects, VEGF can also induce local inflammatory responses and nerve ingrowth by activating VEGFR-1, thereby being associated with the development of discogenic pain (58, 59). Qiu et al. (59) found that, in a rat model of IVD injury, knockout of the VEGFR-1/Flt-1 gene reduced pain sensitivity and simultaneously downregulated the expression levels of pain-related factors surrounding the IVD.

In addition, genetic studies suggest that the VEGF pathway may be associated with susceptibility to IDD. Amaro et al. (60) performed an analysis of VEGF gene polymorphisms in 217 patients with IDD and observed an association between specific polymorphic loci and the IDD phenotype, suggesting that VEGF gene variants may serve as potential clues for risk prediction. A further study by Aydin et al. (61) suggested that this association may influence the degenerative process by regulating angiogenic capacity.

Overall, the role of VEGF in IDD has a certain dual nature. On the one hand, VEGF-mediated angiogenesis may have a certain compensatory significance when local nutritional supply is restricted or the demand for tissue repair increases (35). On the other hand, neovessels within degenerative IVDs are often structurally incomplete and show increased permeability, thereby facilitating the infiltration of inflammatory cells and soluble mediators, inducing local edema, microhemorrhage, and amplification of inflammatory cascades, and subsequently accelerating matrix degradation and promoting the worsening of degeneration (62). Therefore, VEGF should be understood as a key regulatory node linking hypoxic responses, inflammatory amplification, matrix remodeling, and neurovascular invasion, rather than as a unidirectional pathogenic factor.

##### 3.2.1.2 Fibroblast growth factor

The FGF family currently comprises 23 members and regulates cell growth, differentiation, survival, and migration; abnormalities in its signaling are closely associated with a variety of diseases, including cancer, skeletal disorders, metabolic disturbances, and developmental defects (63, 64). Studies have shown that FGF signaling participates in the progression of IDD through multiple mechanisms. Lu et al. (65) demonstrated in a rabbit model of IDD that overexpression of FGF18 significantly downregulated the expression levels of MMP-3, ADAMTS-5, and the pro-apoptotic protein Bax, while upregulating the level of the anti-apoptotic protein Bcl-2, accompanied by a reduction in the proportion of cleaved caspase-3-positive cells. These findings suggest that FGF18 may alleviate the degenerative process by inhibiting apoptosis and matrix degradation.

In the regulation of angiogenesis, FGF signaling can bind to fibroblast growth factor receptors (FGFRs) on the surface of endothelial cells, thereby promoting endothelial cell proliferation and migration and inducing tube-like structure formation in a three-dimensional culture environment (14, 66). Notably, compared with acidic FGF, basic FGF exhibits stronger pro-angiogenic activity in specific experimental systems (66). In addition, FGF signaling can regulate the expression of various proteases, such as MMPs, thereby affecting the balance between ECM degradation and remodeling and providing a suitable microenvironment for the inward growth of neovessels (14, 66).

In degenerative IVD tissues, Tolonen et al. (67) found through immunohistochemical analysis that basic FGF was widely expressed in degenerative tissues, with its immunoreactivity mainly localized to neovascular structures and cells adjacent to AF injury regions. This spatial distribution pattern suggests that FGF signaling may participate in the remodeling of the degeneration-associated microenvironment, particularly playing a role in the process of abnormal vascular proliferation. However, current evidence remains insufficient to establish a causal relationship between FGF expression and vascular invasion; whether it is an active driver of angiogenesis or a concomitant phenomenon after tissue injury still needs to be verified through interventional studies such as gain- and loss-of-function experiments.

##### 3.2.1.3 Platelet-derived growth factor

PDGF is a class of polypeptide growth factors that can be secreted by platelets, macrophages, endothelial cells, and certain mesenchymal cells. By binding to its receptors, PDGFRα or PDGFRβ, PDGF participates in multiple biological processes, including cell proliferation, migration, vascular-related remodeling, and tissue repair (68). PDGF exerts certain protective effects in the early stage of degeneration and can ameliorate degeneration-associated phenotypes by regulating the MAPK pathway and inhibiting pyroptosis, among other mechanisms (69). However, recent single-cell transcriptomic studies suggest that the PDGF–PDGFR signaling axis may be associated with degeneration-related vascularization. Based on single-cell RNA sequencing, Sun et al. (70) identified a lum^+^ cell subpopulation associated with AF angiogenesis in degenerative IVDs. Through bioinformatic analysis and immunofluorescence colocalization, PDGFRα was found to be one of its specific markers, suggesting that PDGFRα-mediated intercellular communication may participate in the formation or remodeling of vascular-related structures in the AF region. However, the current evidence is mainly based on transcriptomic-level association analyses, and the causal relationship between this signaling axis and actual vascular invasion still requires further validation through spatial transcriptomics, lineage tracing, or functional blockade experiments.

##### 3.2.1.4 Transforming growth factor-β

TGF-β is a class of multifunctional cytokines that is widely involved in physiological processes such as embryonic development, maintenance of tissue homeostasis, injury repair, and regulation of the immune microenvironment (71). In IDD, the role of TGF-β1 has a dual nature: on the one hand, it can exert tissue-protective effects under certain conditions through the Smad2/3 pathway (72); on the other hand, some studies suggest that it can promote NP cell apoptosis, inflammatory responses, and ECM degradation through the NF-κB pathway, thereby accelerating the degenerative process (73). Therefore, the biological effects of TGF-β1 in IDD may depend on the disease stage, the state of the local microenvironment, and the activation context of downstream signaling pathways.

In addition to participating in degeneration-related cellular responses, TGF-β1 is also closely associated with angiogenesis and osteochondral remodeling. Wang et al. (74) found that downregulation of TGF-β1 promoted osteoclast differentiation, leading to reduced bone mass in the vertebral body and CEPs, while also inducing abnormal bone-related angiogenesis in the CEPs and growth plate and triggering endplate ossification. This, in turn, disrupted the semipermeable barrier function of the growth plate, disturbed IVD nutrient supply and metabolic exchange, reduced the number of NP cells, and ultimately led to IDD. The study further pointed out that TGF-β1 can also regulate the expression of osteogenesis-related H-type vessels and molecules such as VEGF, suggesting a potential mechanism from the perspective of coupling between angiogenesis and osteochondral remodeling.

In addition, Cui et al. (75) reported that TGF-β1 can promote the upregulation of Ang-2, thereby aggravating inflammatory responses and fibrosis in degenerative NP cells, suggesting that it may participate in remodeling of the degenerative microenvironment through Ang-2, a regulatory node associated with vascular stability. Taken together, existing studies suggest that the role of TGF-β1 in IDD is more likely to be characterized by context-dependent regulatory effects: it may protect IVD homeostasis by maintaining endplate structure and inhibiting aberrant vascularization, while under specific signaling contexts it may also amplify inflammatory and fibrotic responses.

##### 3.2.1.5 Angiopoietin-2

Ang-2 is a member of the angiopoietin (Ang) family and interacts with the Tie2 receptor to regulate vascular stability; its expression is commonly observed in sites with relatively active vascular remodeling (76). Ang-2 is primarily stored in Weibel–Palade bodies of endothelial cells and is rapidly released in response to stimuli such as inflammation, hypoxia, or changes in shear stress (76, 77). Its function depends on the VEGF context: in the absence of VEGF, Ang-2 disrupts the contact between vascular endothelial cells and the matrix by antagonizing the stabilizing effect of Ang-1, thereby inducing vascular regression; in the presence of VEGF, Ang-2 synergistically promotes vascular endothelial cell migration and proliferation (76, 78). Ang-2 can also upregulate IL-6 and TNF-α through inflammation-related pathways such as NF-κB and increase the expression of MMPs, suggesting that it may simultaneously participate in vascular-related alterations, inflammatory responses, and matrix degradation (78).

Existing studies indicate that Ang-2 participates in the progression of IDD through multiple mechanisms, including the regulation of vascular alterations. Ao et al. (79) found that mechanical stress can affect the HIF-1α/NF-κB signaling axis by regulating Ang-2, thereby coupling angiogenesis with matrix degradation. Cui et al. (75) proposed that TGF-β1 can aggravate inflammation and fibrosis via Ang-2. Yu et al. (80) reported that downregulation of Ang-2 by lncRNA GAS5 can reduce NP cell apoptosis and inhibit degeneration. Taken together, these studies indicate that Ang-2 participates in processes such as regulation of vascular stability, amplification of inflammation, and matrix remodeling in IDD, and it can be regarded as a regulatory molecule linking mechanical stimuli, inflammatory signaling, and tissue remodeling. However, most existing evidence is derived from associative observations or single-pathway studies, and the specific hierarchical role and causal status of Ang-2 in IDD still need to be further clarified through temporal studies and functional intervention experiments.

#### 3.2.2 Upstream regulatory factors coupled with growth factors

##### 3.2.2.1 Hypoxia-inducible factor

HIF is a key transcription factor that is stably activated in hypoxic microenvironments, among which HIF-1α is widely expressed in the vast majority of mammalian tissue cells and plays a central regulatory role in cellular adaptation to hypoxia (81). Following the onset of IDD, CEP function declines, resulting in insufficient oxygen supply in local regions and the formation of a hypoxic microenvironment, which in turn activates the HIF signaling pathway and regulates the expression of genes related to angiogenesis, energy metabolism, and cell survival (82, 83). Among these, VEGF is one of the classical downstream target genes of HIF-1α (84). The stabilized nuclear HIF-1α dimer can directly bind to the functional hypoxia response element (HRE) upstream of the promoter region of the human VEGF gene, and this binding event represents a key rate-limiting step in the initiation of VEGF gene transcription (85, 86).

Simona et al. (15) pointed out that HIF-1α is specifically and significantly upregulated in severely degenerated human IVD tissues, and that the hypoxia-response signaling mediated by HIF-1α is a key mechanism driving aberrant angiogenesis in late-stage degeneration. Activation of HIF can promote the expression and secretion of VEGF, thereby inducing neovascularization within the IVD (83). Kwon et al. (87) confirmed that, in addition to VEGF, activated HIF-1α in IVD cells can also upregulate the expression of inflammatory cytokines such as IL-6 and IL-8, as well as angiogenic factors such as vascular cell adhesion molecule (VCAM). Among these, VCAM can reduce the stability of endothelial cell junctions through the NF-κB pathway, stimulate endothelial cell proliferation and migration, and promote microvessel formation. Meanwhile, HIF-1α can also upregulate the expression of matrix-degrading enzymes such as MMP-1 and MMP-3, while downregulating the expression of tissue inhibitor of metalloproteinase-1 (TIMP-1) and TIMP-2, thereby enhancing the ability of microvessels to penetrate into the deep NP tissue and accelerating the progression of IDD. Interestingly, Liu et al. (88) found that overexpression of HIF-1α can upregulate the expression of NOTCH1, its ligand JAGGED1, and the downstream target gene HES1, while also increasing the expression of type II collagen and aggrecan in human NP cells, which further reflects the complexity of the role of HIF-1α in IDD.

In studies of bone metabolic diseases, HIF-1α is regarded as a key molecule coupling angiogenesis with osteogenesis, and it bidirectionally regulates the differentiation of macrophages into osteoclasts under different conditions (89). Song et al. (90) further confirmed that the VEGF/AKT/mTOR signaling pathway is a key regulatory axis through which HIF-1α mediates the coupling between osteogenesis and angiogenesis, providing an important theoretical basis for simultaneously targeting bone metabolism and angiogenesis in IDD in subsequent studies.

##### 3.2.2.2 P53

p53 is a transcription factor encoded by the TP53 gene. It is activated under stress conditions such as DNA damage, oxidative stress, hypoxia, and abnormal mechanical loading, and maintains genomic stability by inducing cell cycle arrest, apoptosis, or senescence (91–93). Classical theory holds that wild-type p53 is a potent inhibitor of angiogenesis, and its mechanisms include the transcription-dependent upregulation of endogenous anti-angiogenic factors, such as thrombospondin-1 and BAI1 (94); the transcriptional repression of pro-angiogenic factors, such as VEGF (94); and the promotion of HIF-1α degradation through binding to the HIF-1α protein (95). In addition, p53 can directly induce endothelial cell apoptosis through the mitochondrial pathway (96).

However, Lu et al. (97) examined pathological sections from 156 postoperative patients with lumbar disc herniation (LDH) and found that the positive expression rates of VEGF and p53 in degenerative IVD tissues were as high as 73.42% and 58.97%, respectively. Moreover, the expression rates of both molecules in tissues with vascular infiltration were significantly higher than those in tissues without infiltration, suggesting that p53 is positively correlated with VEGF in IDD and that the two may jointly promote neovascularization in IDD.

The above phenomena may be collectively explained by the following mechanisms. First, degenerative IVDs remain in a prolonged state of oxidative stress and DNA damage (98), which may provide a basis for TP53 gene mutations. Mutant p53 loses its inhibitory function and instead acquires pro-angiogenic activity—it can synergistically activate VEGF expression with transcription factors, enhance HIF-1α stability, and regulate miRNA networks to promote angiogenesis (99). Therefore, the high level of p53 detected in IDD may include a high proportion of mutant p53, which may synergize with VEGF to drive neovascularization. Second, even if a fraction of wild-type p53 is present, its inhibitory effect may be overwhelmed by strong pro-angiogenic signals in the degenerative microenvironment. Under severe hypoxic conditions, HIF-1α is markedly stabilized and activated (15), and its ability to drive VEGF expression exceeds the inhibitory threshold of p53; meanwhile, inflammatory cytokines such as TNF-α and IL-1β continuously activate the NF-κB pathway and upregulate VEGF expression (99, 100). Under the combined effects of hypoxia and chronic inflammation, the anti-angiogenic program of p53 cannot be effectively executed, whereas the pro-angiogenic signals mediated by HIF-1α and NF-κB become dominant (15). Third, sustained activation of p53 drives a large number of IVD cells toward a senescent state (99). Senescent cells secrete high levels of VEGF and other pro-angiogenic factors, such as IL-8, through the senescence-associated secretory phenotype (SASP) (99, 101). These factors act in a paracrine manner on endothelial cells invading the IVD margins, stimulating their proliferation and migration (99).

### 3.3 Inflammatory responses and chemokines

#### 3.3.1 Immune cells and inflammatory responses

Normal IVD tissue exists in a relatively immune-privileged state. After the onset of IDD, autoantigens in the NP are exposed, triggering immune recognition and inflammatory responses (9). Meanwhile, NP cells and AF cells shift toward a pro-inflammatory phenotype, activating pathways such as NF-κB and MAPK and inducing the expression of multiple inflammatory mediators (102, 103). In addition, chemokines secreted by IVD cells, such as CCL2/MCP-1, CCL3, and CCL4, further recruit various immune cells and continuously amplify inflammation (104).

Macrophages are the predominant infiltrating immune cells in IDD, and their polarization state directly affects the process of angiogenesis (105, 106). During the early stage of degeneration or the phase of acute injury, high concentrations of pro-inflammatory factors in the microenvironment drive macrophage polarization toward the M1 phenotype (105). M1 cells release large amounts of pro-inflammatory factors, including TNF-α, IL-1β, and IL-6, as well as MMPs (40), and promote angiogenesis through multiple pathways: TNF-α can directly activate the NF-κB pathway to upregulate VEGF expression (40); IL-1β can induce the transcriptional expression of VEGF, NGF, and BDNF in NP cells, thereby synchronously activating pro-angiogenic and pro-neurogenic programs (107, 108). IL-1β secreted by M1 cells can also activate the IRE1 pathway in NP cells and upregulate catabolic factors such as MMP-13 and ADAMTS-5, thereby degrading the ECM barrier and providing physical space for vascular sprouting and invasion (109). In addition, M1 macrophages release chemokines such as CCL2 and CCL3 to form a positive feedback loop, continuously recruiting more monocytes and amplifying angiogenic signals (110, 111).

As IDD progresses, the expression of anti-inflammatory factors such as IL-4, IL-10, and TGF-β increases, driving macrophage polarization toward the M2 phenotype (105). M2 cells exert dual effects in the regulation of angiogenesis by secreting anti-inflammatory factors such as TGF-β and IL-10: on the one hand, low concentrations of TGF-β can activate the Smad2/3 pathway, promote ECM synthesis, and indirectly limit excessive vascular invasion (112, 113); on the other hand, M2 macrophages themselves are also an important source of VEGF, and their secreted CHI3L1 can upregulate the expression of MMP-3 and MMP-9 by activating the IL-13Rα2/MAPK pathway, thereby promoting vascular endothelial cell migration (113, 114). In late-stage degeneration, M2 macrophages are closely associated with fibrosis and endplate remodeling, and the TGF-β they secrete can promote perivascular fibrosis, alter the local microenvironment, and affect the structure and function of neovessels (105).

Mast cells are another important class of immune cells in degenerative IVDs, and their number in painful degenerative IVD tissues is significantly higher than that in painless autopsy controls (115). As first responders to tissue injury, mast cells can initiate local angiogenic responses within minutes through the release of preformed mediators during degranulation (116). Tryptase promotes endothelial cell proliferation and tube-like structure formation by activating the protease-activated receptor-2 (PAR2) signaling pathway and upregulating the expression of IL-6, TNF-α, and VEGF. VEGF released by mast cells can directly act on VEGFR-2 on the surface of endothelial cells, activate the downstream PI3K/Akt pathway, and induce endothelial cell migration and vascular formation. In addition, mast cell-derived NGF can synergize with VEGF to promote concomitant neurovascular ingrowth, thereby providing a structural basis for discogenic pain (115).

There is a synergistic amplification effect between mast cells and macrophages. Chemokines released by mast cell degranulation, such as CCL2/MCP-1, can further recruit macrophages to degenerative sites and sustain inflammatory responses; conversely, IL-1β and TNF-α secreted by macrophages can activate neighboring mast cells, forming a positive feedback loop between immune cells (115, 117). In addition, bidirectional regulation also exists between mast cells and IVD cells. Soluble factors derived from degenerative NP cells can induce mast cell degranulation; in turn, mast cell-conditioned medium can significantly upregulate the expression of IL-6, ADAMTS5, and CCL2/MCP-1 in NP cells and CEPs, promote catabolism, and create conditions for vascular invasion (110, 115). Furthermore, soluble factors derived from AF cells may suppress the pro-inflammatory and pro-angiogenic phenotype of mast cells, suggesting a unique barrier function of the AF in maintaining IVD immune homeostasis (115).

In addition to macrophages and mast cells, infiltration of activated B cells, CD4^+^/CD8^+^ T cells, and NK cells can also be observed in some degenerative IVD tissues (44). IL-17 can be secreted by various T cells (118), and it can activate the JAK/STAT signaling pathway and upregulate VEGF expression in NP cells, thereby promoting microvascular invasion in a dose- and time-dependent manner (119). B cells can participate in autoimmune responses by secreting antibodies, thereby indirectly affecting the angiogenic microenvironment (44). Together, these immune cells constitute a complex cell–factor network and release large amounts of inflammatory factors and chemokines, causing IVD tissues to lose their immune-privileged state and accelerating vascular formation.

There is a mutually reinforcing positive feedback relationship between inflammatory responses and angiogenesis. In the early stage of degeneration, immune cells are recruited to IVD tissues and secrete inflammatory cytokines such as IL-1β and TNF-α, thereby inducing AF cells to upregulate the expression of IL-6, IL-8, VEGF, MMP-1, and MMP-3 and creating conditions for angiogenesis (120). After neovessels are formed, chemokines secreted by endothelial cells further recruit more immune cells, and the vasculature serves as a conduit for immune cell infiltration, allowing more monocytes to enter the interior of the IVD (43, 49, 121), thereby forming a coupled cycle of “immune cell infiltration–inflammatory amplification–angiogenesis–tissue degeneration.” Meanwhile, vascular endothelial cells can also affect the catabolic phenotype of AF cells by releasing endothelial microparticles (EMPs), thereby further weakening the ECM barrier (42). In addition, pyroptosis can participate in this process by activating inflammatory mediators such as IL-1β, thereby further promoting microvascular formation and invasion (122).

From the perspective of clinical translation, the immune–vascular coupling mechanism suggests multiple intervention nodes: inhibiting M1 macrophage polarization, such as by blocking the NF-κB and MAPK pathways; promoting phenotypic switching of M2 macrophages, such as by supplementing IL-4 and IL-10; stabilizing mast cell membranes to block degranulation, such as with sodium cromoglycate; antagonizing the PAR2 signaling pathway; and targeting chemokine axes, such as CCL2/CCR2 and SCF/c-Kit. However, it is necessary to avoid simply equating “anti-inflammation” with “treatment” while ignoring disease-stage specificity and the beneficial roles of immune cells.

#### 3.3.2 Chemokines

Chemokines are a class of soluble cytokines that bind to G protein-coupled receptors and mediate the directed migration of immune cells (121). In the IDD microenvironment, in addition to recruiting immune cells, chemokines can also act directly on endothelial cells to participate in angiogenesis (123). To date, upregulated expression of multiple chemokines and their receptors, including members of the CXC and CC families, has been identified in degenerative IVDs, collectively constituting an immune–vascular regulatory network (121).

Stromal cell-derived factor-1 (SDF-1; also known as CXCL12) belongs to the CXC chemokine family, and its canonical receptor is CXCR4 (123). Zhao et al. (124) found that the SDF-1/CXCR4 axis is upregulated in degenerative IVD tissues and that serum SDF-1 levels are positively correlated with Pfirrmann grade and patients’ pain scores. Zhang et al. (123) regulated SDF-1 expression in NP cells and found that, as SDF-1 levels increased, the proliferation and migratory capacity of vascular endothelial cells, as well as *in vitro* tube-like structure formation, were enhanced; this process was partially dependent on activation of the PI3K/Akt signaling pathway. In addition, SDF-1 can also bind to CXCR7, activate the PI3K/Akt signaling pathway, and promote microvascular invasion into the NP (125).

CXCL8 is mainly secreted by macrophages and T lymphocytes, and its expression is increased in degenerative IVDs, where it is associated with pain severity in patients (121). Mechanical stress stimulation can induce NP and AF cells to secrete CXCL8, which participates in the degenerative process by recruiting neutrophils and promoting vascularization (126, 127). CXCL10 is highly expressed in degenerative IVDs (128), and its receptor, CXCR3, is mainly present on Th1 cells and activated T lymphocytes (129). The CXCL10/CXCR3 signaling axis contributes to the maintenance of neuropathic pain through activation of the ERK and p38 pathways and may also mediate the resorption of herniated IVD tissue (130–132).

### 3.4 Notochordal cells

Notochordal cells (NCs) in the human NP begin to decrease after puberty and are gradually replaced by smaller chondrocyte-like cells lacking obvious vacuolar structures, namely the cells commonly referred to as NP cells. As the number of NCs in the NP decreases and their function declines, early degenerative changes may occur in the IVD, including reduced water content and microfissures in the AF. Therefore, the reduction and functional decline of NCs are considered to be potentially associated with the onset of early IDD (133).

Recent studies suggest that NCs participate in maintaining the relatively avascular state of the NP. Cornejo et al. (31) reported that soluble factors secreted by NCs derived from immature NP can significantly downregulate the expression of molecules such as IL-6, IL-8, MMP7, and VEGF in endothelial cells, suggesting that NCs may restrict vascular-associated phenotypes by suppressing pro-inflammatory and pro-angiogenic molecules. After isolating and analyzing the transcriptional characteristics of human NC-like cells, Rodrigues-Pinto et al. (134) found that molecules associated with angiogenesis inhibition, including WISP2, Noggin, and EDN2, as well as IL1RN, which is associated with inflammation antagonism, constitute important components of their transcriptional network, suggesting that NCs may jointly maintain NP microenvironmental homeostasis by simultaneously regulating inflammation and angiogenesis. Another study showed that, under hypoxic conditions, NCs can inhibit the angiogenic capacity of endothelial cells, accompanied by downregulation of vascular-related signals such as VEGF and FAK/F-actin/PDGF (135).

In addition to soluble factors, the exosomal pathway is also considered to participate in the regulation of angiogenesis within the IVD by NCs. Sun et al. (136) found that, under a pressure load of 0.5 MPa, NC-derived exosomes can inhibit neomicrovascularization within the NP, and this effect is mainly mediated through the miR-140-5p/Wnt/β-catenin pathway. Under conditions of abnormal pressure loading, NCs exhibit greater stress resistance than NP cells; therefore, their associated biological effects are more pronounced. Further studies suggest that miR-140-5p can significantly downregulate the activities of MMP-2 and MMP-7, thereby inhibiting MMP-mediated matrix degradation and restricting microvascular invasion. At the same time, Wnt/β-catenin-related pathways play important roles in endothelial cell proliferation, migration, and microvascular formation, and are also involved in MMP regulation; NC-derived exosomes can inhibit the activation of this pathway. In addition, NCs also exert an inhibitory effect on nerve ingrowth during the progression of IDD (137).

However, the findings of de Vries et al. (138) differ from those described above. They found that notochordal cell-conditioned medium (NCCM) and native notochordal cell-derived matrix (NCM) did not exhibit stable inhibitory effects on angiogenesis or neurite outgrowth and that, under certain experimental conditions, NCCM could even promote angiogenesis. These discrepancies suggest that the anti-angiogenic and anti-nerve ingrowth effects associated with NCs may be context-dependent, and that the results may be influenced by multiple factors, including donor age, species origin, tissue sampling site, culture oxygen tension, the preparation procedure for conditioned medium, and the status of recipient cells.

Overall, NCs are an important cellular component in maintaining NP microenvironmental homeostasis. Their reduction or functional decline may weaken the local constraint on pro-angiogenic and pro-nerve ingrowth signals, thereby increasing susceptibility to pathological vascular and neural ingrowth (133). In the field of biological therapy for IDD, the translational application of NCs remains limited by cell sources, difficulties in acquisition, and ethical issues. Compared with direct cell transplantation, strategies based on NC-secreted products, such as exosomes, cell immortalization, or xenogeneic cell substitution may have greater feasibility for early application. Although studies have begun to explore the induced acquisition of NCs and their therapeutic potential, their stable protective effects in *in vivo* animal experiments, specific mechanisms of action, and translational feasibility remain to be further systematically investigated.

### 3.5 MicroRNA

MicroRNAs (miRNAs/miRs) are a class of endogenous small non-coding RNAs that mainly regulate the expression of target genes at the post-transcriptional level and play important roles in the maintenance of cellular phenotypes and the regulation of tissue homeostasis. In recent years, studies have shown that abnormal miRNA expression profiles are closely involved in the molecular pathological process of IDD and regulate degenerative progression by affecting mechanisms such as the hypoxic response, inflammatory response, and angiogenesis (139).

With respect to angiogenesis-related regulation, studies have suggested that certain miRNAs influence vascular-associated phenotypes in degenerative IVDs by modulating different signaling axes. Among them, miR-140-5p has been reported to inhibit the angiogenic phenotype of endothelial cells through the Wnt/β-catenin signaling pathway, and functional intervention experiments have confirmed that it can reduce the level of angiogenesis in degenerative IVDs in mice (136). Sheng et al. (140) found that, after inhibition of miR-21 in a rat IDD model, the levels of HIF-1α and VEGF in NP and AF tissues both decreased, accompanied by an increase in proteoglycan content and a reduction in apoptosis, suggesting that miR-21 may participate in the activation of the HIF-1α/VEGF axis. Wang et al. (141) found that the expression of miR-101-3p was significantly decreased in degenerative NP tissues; they further confirmed that miR-101-3p can directly bind to the 3′ untranslated region of stanniocalcin-1 (STC1) mRNA, inhibit STC1 expression, and thereby reduce microvascular generation and proliferation through the STC1/VEGF/MAPK signaling pathway, thus delaying IDD progression.

### 3.6 The semaphorin family

Semaphorins are a family of secreted or transmembrane proteins that, in addition to their classical role in axon guidance, are also involved in processes such as cell migration, angiogenesis, immune regulation, and bone metabolism (142). In IDD, class III semaphorins are closely associated with vascular and neural ingrowth and may participate in the molecular barrier that maintains the “relatively aneural/avascular” state of the IVD (143). As an anti-angiogenic factor, Sema3A is significantly downregulated in degenerative AF, lowering the local inhibitory threshold against neurovascular ingrowth and thereby promoting its abnormal inward extension along the AF (143, 144). Binch et al. (145) observed upregulated expression of Sema3C, Sema3D, and their receptor neuropilin-2 in degenerative IVD samples with evidence of neurogenesis and angiogenesis. Among these, Sema3C is considered to have a potential chemoattractant-like effect and may participate in regulating the inward growth of blood vessels and nerve fibers; Sema3D, by contrast, is more likely to be biased toward neurotaxis-related processes, with a relatively limited association with angiogenesis.

### 3.7 Tissue inhibitors of metalloproteinases

TIMPs are the principal endogenous inhibitors of MMPs and participate in processes such as ECM remodeling and angiogenesis in IDD. HIF-1α can downregulate the expression levels of TIMP-1 and TIMP-2, thereby enhancing the penetrative capacity of microvessels into the deep NP tissue (87). TIMP-3 can inhibit endothelial cell proliferation and migration by binding to VEGFR-2 or blocking the binding of VEGF to VEGFR-2, while also reducing the secretion of the inflammatory cytokines TNF-α, IL-1β, and IL-6, delaying ECM degradation, and exerting anti-angiogenic effects (39, 146). He et al. (39) found that TIMP3 expression was decreased in degenerative IVDs from patients with IDD, and that upregulation of TIMP3 could significantly reduce the levels of inflammatory cytokines (TNF-α, IL-1β, and IL-6) and VEGF in degenerative IVD tissues, thereby alleviating pathological IVD degeneration and inhibiting neovascularization.

### 3.8 Other regulatory factors

The anti-aging protein Klotho decreases with age, and its low expression has been observed in various age-related diseases, including IDD (147). Yi et al. (148) found that exogenous supplementation with Klotho can inhibit the Rac1/PAK1/MMP-2 signaling axis, downregulate the expression of angiogenesis-related molecules such as VEGF, CD34, and MMP-2/9, and reduce the endothelial cell migratory response induced by conditioned medium from senescent NP cells. In addition, Klotho is also considered an endogenous inhibitor of the Wnt/β-catenin signaling pathway; decreased Klotho expression may relieve the inhibition of angiogenesis-related transcriptional programs through this pathway, thereby enhancing the tendency toward vascularization in the degenerative microenvironment (149).

Heat shock protein 90 (HSP90) is an important regulatory node linking inflammation, fibrosis, and angiogenesis (150, 151). Zhang et al. (150) found that fibrotic NP cell clusters are closely associated with the angiogenic process and that macrophages, especially the M2 subtype, induce NP cells to express the fibrosis marker CEMIP as well as VEGF-A and FGF2 through secreted factors, thereby promoting endothelial cell migration and angiogenesis. Further functional experiments showed that intervention with the HSP90 inhibitor 17-AAG or specific siRNA not only significantly reversed the macrophage-induced fibrotic phenotype and inhibited the activation of key angiogenesis-related pathways (RhoA-ROCK and Akt) *in vitro*, but also effectively alleviated the degree of NP fibrosis and blocked pathological vascular ingrowth in *in vivo* animal models.

Tenomodulin (Tnmd) is a specific marker of avascular tissues such as tendons, ligaments, and the IVD, and also has significant anti-angiogenic effects (152). Lin et al. (153) found that Tnmd is mainly localized in the ECM of the outer AF. In the absence of Tnmd, the expression of CD31, P65, MMP-3, and MMP-9 is significantly upregulated, accompanied by an increase in macrophage content and enhanced migration of human umbilical vein endothelial cells.

### 3.9 The permissive role of the systemic pathological microenvironment in neovascularization

Obesity is an independent risk factor for IDD, and a higher body mass index (BMI) is positively correlated with the prevalence and severity of IDD (154, 155). As an endocrine organ, adipose tissue can secrete various adipokines that participate in the regulation of inflammation and angiogenesis (156). Among these, fatty acid-binding protein 4 (FABP4) is highly expressed in adipocytes and participates in fatty acid transport and metabolism (157). Han et al. (158) found that obesity upregulates FABP4 expression by activating the mTORC1 signaling pathway, promotes the accumulation of advanced glycation end products (AGEs), activates the AGEs-RAGE/NF-κB signaling axis, and thereby induces an imbalance in ECM synthesis in NP cells, characterized by decreased type II collagen and aggrecan and increased MMP expression. Meanwhile, it upregulates VEGF to promote aberrant neovascularization as well as endothelial cell migration and tube formation, thereby accelerating IDD progression. Genetic knockout and pharmacological inhibition of FABP4 can block the above cascade, restore ECM homeostasis, inhibit pathological angiogenesis, and ameliorate the degree of IDD in obese mice. These findings reveal the critical role of the mTORC1-FABP4-AGEs-RAGE/NF-κB pathway in obesity-driven IDD and provide a potential therapeutic target for IDD in obese populations.

Diabetes mellitus, particularly type 2 diabetes mellitus, can accelerate the progression of IDD (159). As the principal route for nutrient supply to the IVD, the structural and functional integrity of the CEPs is critical for maintaining IVD homeostasis (25). A high-glucose environment can induce excessive ROS accumulation in CEP cells, leading to loss of mitochondrial membrane potential, dysfunction of the respiratory chain, and impaired ATP synthesis, and subsequently activating the caspase cascade through the mitochondrial pathway to induce chondrocyte apoptosis (160). Meanwhile, high glucose-induced oxidative stress can upregulate the expression of pro-angiogenic factors such as VEGF by activating the NF-κB and HIF-1α signaling pathways (161). ROS itself can also act as a signaling molecule to promote the release of inflammatory cytokines through activation of the MAPK pathway (162), thereby forming a cascade amplification effect of “oxidative stress–inflammation–angiogenesis.” Quan et al. (17) established a mouse model of type 2 diabetes mellitus using a high-fat diet combined with streptozotocin and found that hyperglycemia induced upregulated expression of the vascular endothelial marker CD31 in the CEPs, aggravated peripheral vascular ingrowth, increased infiltration of pro-inflammatory monocytes/macrophages (F4/80^+^/CD16/32^+^), and elevated levels of the inflammatory cytokines IL-1β and TNF-α. Ultrastructural analysis showed nuclear senescence, mitochondrial abnormalities, and disordered collagen fiber arrangement in CEP chondrocytes. These results confirm that hyperglycemia accelerates IDD progression by inducing oxidative stress and mitochondrial damage, thereby triggering CEP cell apoptosis and inflammatory responses, while simultaneously promoting vascular ingrowth and structural destruction of the CEP through upregulation of pro-angiogenic signaling.

The relationship between osteoporosis and IDD remains controversial, but osteoprotegerin (OPG) plays a key role in their association (57). As a decoy receptor for receptor activator of NF-κB ligand (RANKL), OPG competitively binds to RANKL and blocks its interaction with receptor activator of NF-κB (RANK), thereby inhibiting osteoclast differentiation, activation, and bone resorptive activity (163, 164). In the absence of OPG, the RANKL/RANK signaling pathway remains persistently activated, promoting the differentiation of osteoclast precursors into mature osteoclasts while enhancing the bone-resorbing function of mature osteoclasts (164, 165). Li et al. (57) found that, in OPG knockout mice with severe osteoporosis, the expression levels of CD31, CD34, and vascular endothelial cadherin in the CEP were significantly upregulated, inducing the migration of endothelial cells, hematopoietic cells, and osteoblasts toward the CEP; the expression of inflammatory cytokines such as TNF-α and IL-1β was also upregulated, which further stimulated VEGF expression, promoted neovascularization, and accelerated IDD progression. Notably, osteoclast activation not only leads to bone resorption, but also enables the degradation of ECM through the secretion of MMPs and cathepsin K (166), thereby releasing matrix fragments with pro-angiogenic activity (43). Meanwhile, osteoclasts themselves can also secrete pro-angiogenic factors such as VEGF (167), forming a coupling of “bone resorption–angiogenesis.” These findings suggest that OPG deficiency may participate in neovascularization within the CEP through two pathways: directly upregulating VEGF and indirectly inducing inflammatory responses, whereas the coupling mechanism between osteoclast activation and angiogenesis is a key link connecting osteoporosis and IDD.

**Figure 3:**
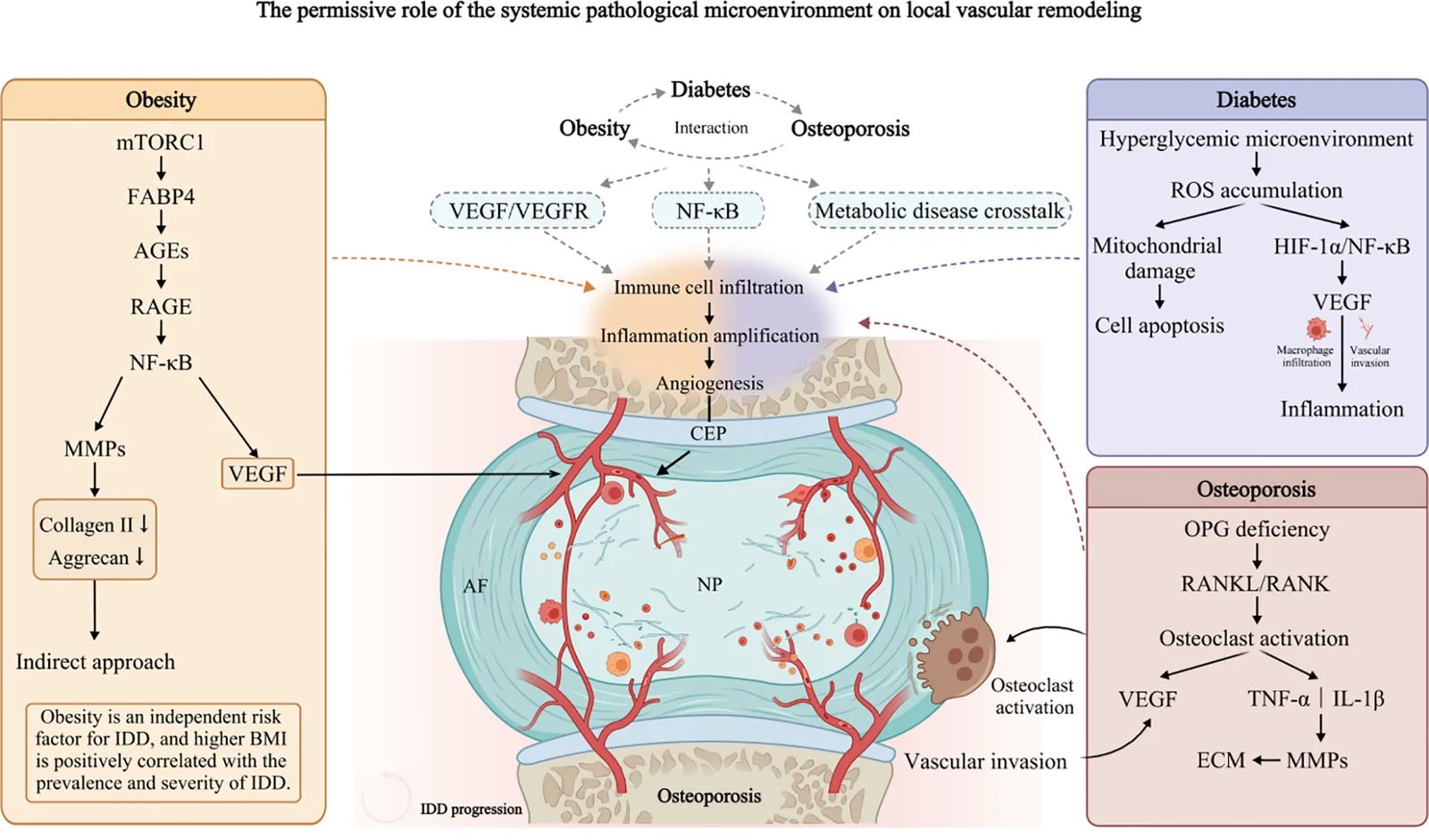
The permissive role of the systemic pathological microenvironment on local vascular remodeling. Obesity, diabetes, and osteoporosis generate a permissive systemic pathological microenvironment that promotes local vascular remodeling in the degenerating intervertebral disc (IVD). In obesity, activation of the mTORC1/FABP4/AGEs/RAGE/NF-κB pathway increases matrix metalloproteinases (MMPs) and vascular endothelial growth factor (VEGF), resulting in extracellular matrix degradation characterized by reduced collagen II and aggrecan. In diabetes, hyperglycemia induces reactive oxygen species (ROS) accumulation, mitochondrial damage, and apoptosis, while activating HIF-1α/NF-κB/VEGF signaling to enhance macrophage infiltration, inflammation, and vascular invasion. In osteoporosis, OPG deficiency activates RANKL/RANK signaling and osteoclast activity, thereby increasing VEGF, TNF-α, IL-1β, and MMP-mediated matrix degradation, which further facilitates vascular ingrowth. Crosstalk among these metabolic disorders amplifies VEGF/VEGFR signaling, NF-κB activation, immune cell infiltration, and inflammatory responses, ultimately promoting angiogenesis in the cartilaginous endplate (CEP) region and vascular invasion into the annulus fibrosus (AF) and nucleus pulposus (NP), thereby accelerating IVD degeneration.

Taken together, the above studies indicate that microenvironmental alterations under conditions of obesity, diabetes mellitus, and osteoporosis can all participate in the pathological progression of IDD by promoting neovascularization within the CEP. Although the initiating mechanisms differ among these three pathological conditions, they all converge on a common pathway of “immune cell infiltration–inflammatory amplification–angiogenesis” (**Figure 3**). Meanwhile, potential interactions exist among these three pathological conditions: obesity and diabetes mellitus often coexist, and hyperglycemia and hyperlipidemia synergistically exacerbate oxidative stress and inflammatory responses (168); patients with osteoporosis are often accompanied by age-related metabolic disorders, and metabolic inflammation can also aggravate bone loss (169, 170). This multifactorial additive effect suggests that IDD patients with concomitant metabolic diseases require stratified intervention strategies distinct from those for patients with simple degeneration. In addition, whether these three pathological conditions mediate CEP vascularization through common downstream signaling pathways, such as the VEGF/VEGFR axis and the NF-κB signaling axis, warrants further investigation. From the perspective of therapeutic strategies, for IDD patients with concomitant metabolic diseases, regulation of systemic metabolic disorders, such as glycemic control, modulation of lipid metabolism, and intervention in osteoclast activation, should be considered while inhibiting pathological angiogenesis, so as to achieve more precise disease-modifying therapy.

## 4 The lymphatic system in intervertebral disc degeneration

The core function of the lymphatic system is to recover fluid and macromolecules in the interstitial space that are difficult for capillaries to reabsorb, and to transport immune cells and inflammatory mediators, which ultimately return to the venous circulation (19). In most tissues, lymphatic vessels accompany blood vessels, and together they maintain fluid homeostasis and immune surveillance (19, 171). The lymphatic system is closely associated with the containment and resolution of inflammation; impaired lymphatic drainage can lead to the persistence of various inflammatory diseases, whereas enhanced lymphatic return is important for alleviating inflammation and improving the tissue microenvironment (172). Given that inflammation is a core pathological event in IDD (107), the lymphatic system may represent a key therapeutic target.

Compared with the vascular system, the lymphatic system in the IVD has long been understudied. The traditional view, grounded in the work of Rudert (173), Kliskey (174), Kashima (175), and others, holds that the healthy adult IVD lacks a “classical lymphatic vascular network”. This phenomenon was ascribed a certain physiological significance at the time: the IVD possesses relative immune privilege, and limiting contact between the proteoglycan-rich matrix components of the nucleus pulposus and the systemic immune system may reduce the risk of autoimmune responses (176). Accordingly, under physiological conditions, the clearance of metabolic waste from the IVD has long been considered to depend on diffusion toward capillaries in the cartilaginous endplate and outer annulus fibrosus (8–10). However, recent evidence suggests that IVD-associated lymphatic structures may exhibit a conditional presence that depends on anatomical boundaries and pathological states, which is particularly evident in the outer AF, the peridiscal connective tissue, and in lesions that breach these boundaries (171).

### 4.1 Traditional histological understanding: absence of a classical lymphatic network

Using lymphatic endothelial 5′-nucleotidase activity as a marker and laminin immunohistochemistry to distinguish vascular structures, Rudert et al. (173) systematically examined lumbar IVD autopsy samples ranging from fetal specimens to individuals of advanced age (≤90 years). The study found that lymphatic vessels were mainly distributed in the outer AF of individuals younger than 20 years and in the dorsolateral connective tissue adjacent to the IVD across all age groups, whereas no lymphatic signals were detected in the CEPs and NP region. In terms of spatial distribution, lymphatic structures usually appeared in regions where blood vessels were present, suggesting that lymphatic drainage of IVD-associated tissues may depend on the peridiscal vascularized connective tissue pathway.

Kliskey et al. (174) used excised NP specimens from IVD protrusion and autopsy thoracolumbar IVD samples, and performed localization analysis using an immunohistochemical panel combining lymphatic vessel endothelial hyaluronan receptor 1 (LYVE1), podoplanin, and the vascular endothelial markers CD31/CD34. No lymphatic vessels were observed in either the normal adult NP or AF, whereas lymphatic structures were identified in soft tissues such as the peridiscal ligaments. In addition, reparative fibrous tissue ingrowth was commonly observed in IVD protrusion specimens, with LYVE1^+^/podoplanin^+^ lymphatic vessels present in some regions. These findings support the traditional view that the adult IVD lacks lymphatic vessels and are generally interpreted to indicate that, when IVD tissue crosses its original anatomical boundaries and enters the peridiscal soft-tissue environment, lymphangiogenesis occurs as an accompanying response to inflammation and reparative fibrosis.

Kashima et al. (175) further confirmed in a larger set of normal and pathological tissues that lymphatic vessels were generally absent within intact normal vertebral bodies and IVDs, whereas they could be detected in infected or displaced degenerative IVD tissue. Intraosseous lymphatic vessels in the vertebral body were mainly observed when lesions breached the cortical bone and extended into the surrounding connective tissue.

These studies reached inconsistent conclusions regarding the presence of lymphatic vessels in the outer annulus fibrosus. This discrepancy may reflect an age-dependent pattern of lymphatic structures in this region, with lymphatic vessels undergoing regression or even disappearing with aging. It may also arise from differences in sample composition and detection methods across studies. The specimens differed in age distribution, degree of degeneration, and sampling site, and none of the studies applied Pfirrmann or Thompson grading, leaving substantial heterogeneity in sample characteristics. The detection methods also had limitations: none of the three studies included Prox1, a highly specific marker of lymphatic endothelial cells, making their conclusions less reliable by current standards. Further work is needed to clarify the presence and function of lymphatic vessels within the intervertebral disc.

### 4.2 New evidence at the functional level: lymphangiogenesis and the regulation of degeneration

Zou et al. (171) combined spatial transcriptomics, histological staining, and immunolabeling techniques to characterize the spatial distribution of lymphatic-related markers in healthy and degenerative human IVDs, and conducted functional experiments involving lentivirus-mediated Prox1 overexpression in a rat model of IVD degeneration. The study found that lymphatic-related signals were present in the AF of healthy IVDs, whereas degenerative IVDs showed a reduction in lymphatic vessel number despite a concomitant increase in vascularity. In addition, enhanced lymphangiogenesis was associated with inflammatory cell clearance, preservation of tissue architecture, and attenuation of degeneration. These findings partially revise the traditional view that lymphatic vessels are absent within the adult IVD. On this basis, Zou et al. proposed a mechanistic hypothesis that a reduction in lymphatic-related structures may impair the clearance efficiency of inflammatory cells and inflammatory mediators, thereby promoting persistent inflammation and driving degenerative progression.

In response to the above work, Zhang et al. (177), while recognizing its pioneering significance, proposed the following directions for methodological and mechanistic refinement: the control samples may have been affected by age-related bias; the grading of degeneration (e.g., Pfirrmann/Thompson) should be specified to enable stratified comparisons; expression of markers alone is insufficient to demonstrate the “functional drainage” of lymphatic vessels and requires more direct imaging and functional validation; and further investigation was recommended into the regulatory roles of the classical lymphangiogenic axes, including HIF-1α/VEGF-C/VEGFR-3, in the IVD microenvironment. These considerations reveal the current limitations of IVD-related lymphatic research, namely, that the expression of lymphatic markers is not equivalent to lymphatic vessel formation, and that the “structural presence” of lymphatic vessels is not equivalent to their “functional competence.”

In animal models, Salo et al. (178) observed the expression of VEGF-C, VEGF-D, and their receptor VEGFR-3, which are associated with lymphangiogenesis, in a porcine model of IVD degeneration, and detected a small number of LYVE1-related microstructures in the AF region, suggesting that lymphangiogenic signaling may be activated under degenerative conditions. However, such findings more often reflect “generative potential” at the molecular level, and whether they ultimately lead to effective lymphatic drainage still requires confirmation through functional imaging and *in vivo* tracing techniques.

### 4.3 Potential involvement of lymphatic-related molecular networks in IVD degeneration and related pathological processes

Bioinformatics studies related to lymphatic-immune coupling have provided additional clues. Some studies have inferred changes in immune cell composition during the development of IDD based on transcriptomic data, such as reductions in certain lymphocyte subsets and enhancement of myeloid cell-associated signals, suggesting that the immune microenvironment may undergo remodeling (179). Therefore, as an important route for immune cell migration and homing, the lymphatic system has the theoretical potential to participate in the immune regulation of IDD. In addition, some studies, based on hyaluronan metabolic networks, have proposed that the LYVE1-related axis may be associated with matrix metabolism and waste clearance; however, such inferences remain correlational evidence and should be interpreted with caution and validated through functional experiments (180).

In a systems biology analysis more specifically focused on “lymphatic-associated gene sets,” Lin et al. (181) integrated GEO datasets and applied multiple machine learning algorithms to identify diagnostic signature genes among lymphatic-associated genes (LAGs), construct a predictive model for IDD, and further extend the drug-target and competing endogenous RNA (ceRNA) networks. The value of such work lies in providing a testable set of candidate genes and stratification tools for the hypothesis that “lymphatic-immune-related molecular networks may be involved in IDD.” However, these conclusions still require validation in independent cohorts, spatial localization, and functional experiments to avoid misinterpreting “correlational features” as “mechanistic drivers.”

**Figure 4:**
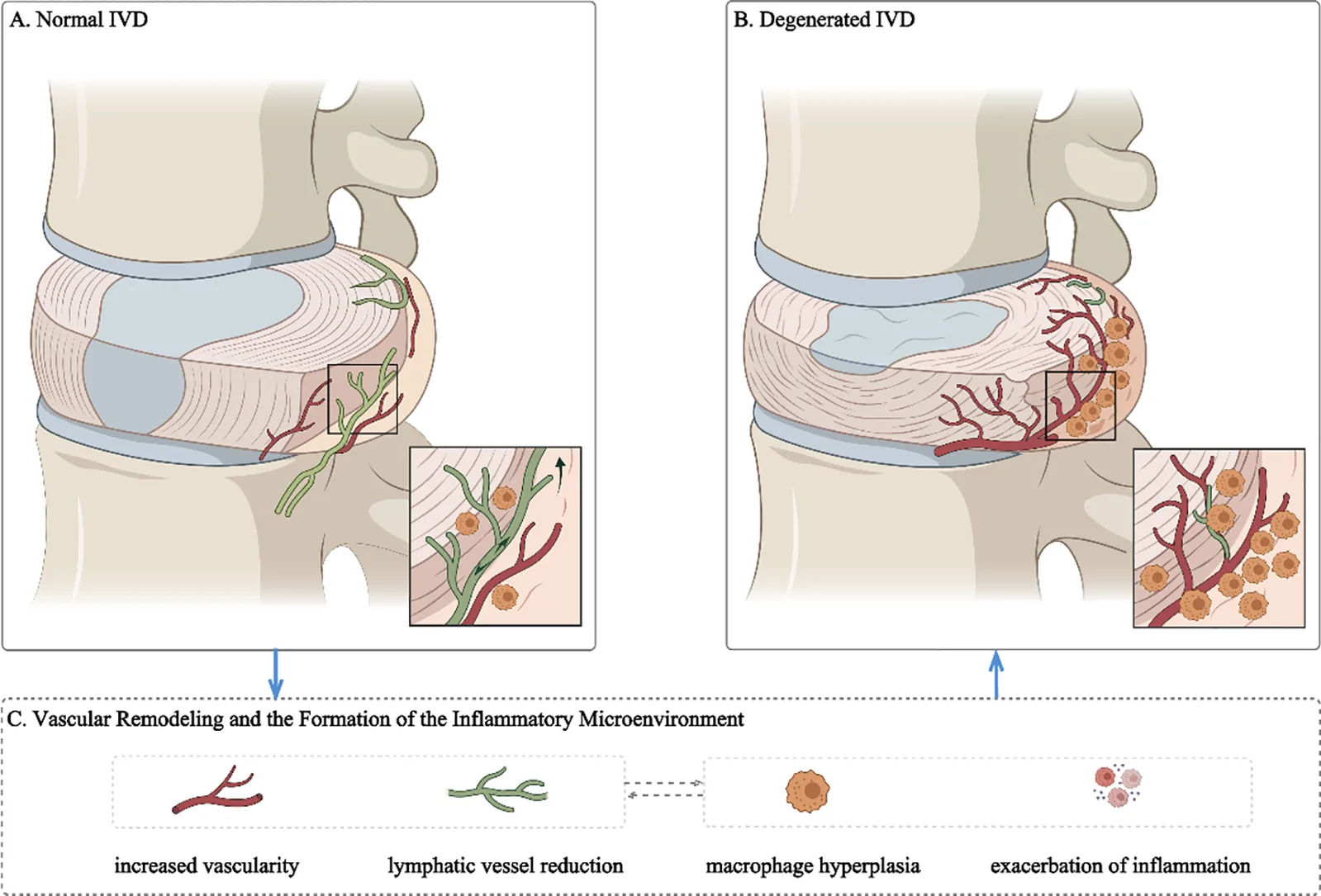
Lymphatic vessel model in intervertebral disc degeneration. **(A)** In the normal intervertebral disc (IVD), blood vessels and lymphatic vessels are primarily restricted to the peripheral region, contributing to tissue homeostasis and immune regulation. **(B)** In the degenerated IVD, pathological vascular remodeling is characterized by increased vascular ingrowth and inflammatory cell infiltration, whereas lymphatic vessels are reduced. **(C)** The combination of increased vascularity, lymphatic vessel reduction, macrophage hyperplasia, and aggravated inflammation promotes the establishment of a pro-inflammatory microenvironment and accelerates the progression of IVD degeneration.

Taken together, the available evidence suggests that the IVD lymphatic system may conform to the following working model (**Figure 4**): under physiological conditions with intact anatomical boundaries, the NP and CEP regions largely lack typical lymphatic structures, whereas the peridiscal connective tissue and the outer AF may perform limited lymphatic-related functions. During degeneration, inflammatory repair, or tissue breaching of anatomical boundaries, lymphatic structures and signaling axes may be induced to undergo remodeling.

Future studies should not be limited to the morphological controversy over “whether lymphatic vessels exist within the IVD,” but should instead further dissect their functional status, including inflammatory drainage and immune regulation, as well as the upstream core regulatory mechanisms. In osteoarthritis and rheumatoid arthritis, impaired lymphatic drainage can lead to the local accumulation of inflammatory factors and matrix-degrading enzymes (MMPs and ADAMTS), forming a vicious cycle of “sustained inflammation-tissue degradation-accelerated disease progression” (172). In IDD, reduced lymphatic function may accelerate the degenerative process through similar mechanisms: on the one hand, decreased lymphatic drainage weakens the clearance efficiency of pro-inflammatory factors (IL-1β and TNF-α) and chemokines (CCL2 and CCL5), thereby maintaining persistent activation of inflammatory signaling; on the other hand, downregulation of the VEGF-C/VEGFR-3 signaling axis may restrict compensatory lymphangiogenesis, further impairing local immune surveillance and waste clearance capacity (172, 182). This functional defect may form pathological coupling with aberrant angiogenesis: neovessels provide infiltration routes for inflammatory cells, whereas impaired lymphatic drainage hinders inflammation resolution, and together they constitute a positive feedback loop of inflammatory amplification. Therefore, the dynamic reduction of lymphatic-related structures during IDD progression may represent a direction requiring further investigation in future studies.

In addition, on the basis of standardized detection criteria and clearly defined sample age and degeneration grading, further studies are still needed to elucidate the dynamic patterns of lymphatic structures across different regions of the IVD and at different stages of disease progression, and to achieve visualized functional assessment of the lymphatic system by means of near-infrared fluorescence tracing (ICG), light-sheet imaging, tissue clearing, and other techniques. From the perspective of clinical translation, targeting the VEGF-C/VEGFR-3 signaling axis to enhance lymphangiogenesis, or applying lymphatic contractility-promoting agents to improve drainage function, may represent new directions for IDD intervention. The functional coupling and dynamic balance between the lymphatic and vascular systems in IDD may become a key entry point for understanding the mechanisms of degeneration and developing combined therapeutic strategies.

## 5 Clinical and pathological significance of alterations in the vascular and lymphatic systems

The pathological remodeling of the vascular and lymphatic systems in the IVD is not an isolated histological phenomenon, but is closely associated with the core clinical manifestations and disease course outcomes of IDD. Available evidence suggests that its clinical significance can be summarized from the following three dimensions. First, it serves as a structural basis for discogenic pain (36, 37, 183). Second, it participates in the resorption of herniated NP tissue (49). Third, it alters the original microenvironment within the IVD and participates in regulating the degenerative process by influencing immune cell infiltration and the local concentrations of inflammatory factors (14–17).

### 5.1 Nerve–vascular complexes: the structural basis of discogenic pain

In the normal adult IVD, blood vessels and nerves are mainly confined to the outermost layer of the AF, whereas the NP and the inner AF are relatively deficient in vascular and neural innervation and therefore usually do not constitute a direct structural basis for the generation of pain signals (36, 37). However, during IDD progression, AF fissures, proteoglycan loss, and microenvironmental alterations can disrupt the IVD barrier, leading to the accompanying inward growth of neovessels and sensory and sympathetic nerve fibers, thereby forming “nerve–vascular complexes” (36, 37, 183). Nerve endings that enter the interior of the IVD can express various nociception-related neuropeptides, such as substance P and calcitonin gene-related peptide (CGRP), and can be activated under the combined effects of abnormal mechanical loading, inflammation, and an acidic microenvironment, thereby generating and transmitting pain signals (184–186). Meanwhile, the density and invasion depth of IVD neurovascularization are associated with the severity of IDD-related pain and functional impairment, which also provides a preliminary explanation for the “inconsistency between the degree of imaging-defined degeneration and pain symptoms” in some patients (37).

At the molecular level, pro-inflammatory factors and pro-angiogenic and pro-neurogenic signals exhibit synergistic amplification effects. IL-1β can simultaneously induce NP cells to upregulate the expression of VEGF, NGF, and BDNF (187). NGF can be expressed by vascular endothelial cells and degenerative IVD cells and, through interactions with pro-angiogenic signals such as VEGF, promote the formation of “nerve–vascular complexes” (48). The aforementioned accompanying growth of nerves and blood vessels may be driven by shared chemokine or growth factor axes, such as SDF-1/CXCR4 and VEGF/NGF interactions (48, 123), but the specific mechanisms remain to be further explored. Meanwhile, inhibition of angiogenesis can reduce behavioral indices related to neuropathic pain, and interventions targeting angiogenesis or matrix degradation may be accompanied by reduced release of pain-related mediators, such as substance P, and improved manifestations of discogenic pain (39, 188). It should be noted that the mechanisms by which angiogenesis inhibition alleviates pain still require further clarification: its effects may arise from blocking accompanying nerve ingrowth, reducing inflammatory cell infiltration, directly decreasing neural sensitization, or the synergistic action of multiple such pathways.

In addition, even when mature vascular structures are not necessarily consistently observed in clinical samples, the expression of pro-angiogenic factors, such as VEGF and FGF-2, may itself reflect a “pro-angiogenic and pro-inflammatory biochemical microenvironment” and may be associated with the risk of persistent postoperative pain and quality-of-life outcomes (189). These findings suggest that a molecular-level angiogenic tendency may serve as a potential indicator for pain prognosis stratification; however, its causal direction and potential confounding factors, such as the degree of degeneration, inflammatory status, patient age, and psychological factors, still require further control and validation in subsequent studies.

### 5.2 Remodeling of the vascular and lymphatic systems and resorption of herniated disc material: potential favorable effects on outcomes

LDH is one of the most common degenerative spinal disorders in clinical practice. Since the 1990s, studies have found that some untreated herniated IVD tissues can decrease in size or even be completely resorbed within several months, with concomitant symptom relief; this phenomenon is referred to as resorption (190). This phenomenon is particularly pronounced in extruded and sequestrated LDH (191). Available evidence indicates that multiple mechanisms are involved in this process (**Table 1**), among which remodeling of the vascular and lymphatic systems plays a key role. Rätsep et al. (49) found that the degree of IVD degeneration and the extent of vascular ingrowth showed concordant trends, and that herniated tissues with richer vascularization had a higher probability of resorption. Tsarouhas et al. (192)further pointed out that the coordinated activation of multiple angiogenesis-related growth factors, including VEGF and TGF-β1, promotes the resorption of herniated IVD tissue. Notably, in the tumor microenvironment, VEGF expression exhibits a highly synchronized spatiotemporal distribution pattern with MMP-3 expression and macrophage infiltration. By promoting endothelial cell proliferation and vascular permeability, VEGF establishes a favorable niche for tumor growth; meanwhile, the proteolytic activity of MMP-3 remodels the ECM and promotes macrophage migration toward hypoxic tumor regions. Through a coordinated regulatory network, these three factors jointly drive tumor angiogenesis and immune evasion (193). Based on the above mechanistic similarities, a similar coordinated regulatory mechanism may also exist during the resorption of herniated discs.

**Table 1:** Mechanisms of intervertebral disc herniation resorption.

Mechanism category	Specific mechanism/process	Key cells/molecules/structures	Primary role	References
Immune-inflammatory response	Autoimmune response	T cells, B cells, autoantibodies	After the NP breaches the AF/posterior longitudinal ligament, it becomes exposed to the immune system, triggering an autoimmune response, antibody production, and initiation of immune-mediated lysis.	(195–197)
	Inflammatory response	Macrophages, TNF-α, IL-1β, IL-6, IL-4, IL-10	Inflammation is a major driving force of resorption. M1 macrophages secrete pro-inflammatory cytokines and initiate clearance, whereas M2 macrophages secrete anti-inflammatory cytokines and contribute to repair. The dynamic balance of inflammatory mediators is critical for successful resorption.	(198, 199)
Vascular and lymphatic systems	Neovascularization	VEGF, bFGF, macrophages	Neovascularization provides pathways for immune cell delivery.	(192)
	Lymphangiogenesis	VEGF-C/D, VEGFR-3, lymphatic endothelial cells	Inflammatory responses upregulate pro-lymphangiogenic factors and induce local lymphangiogenesis. The lymphatic system is responsible for draining inflammatory products and degradation debris, maintaining homeostasis of the inflammatory microenvironment and preventing excessive tissue damage.	(194)
ECM degradation and cell death	ECM degradation	MMP-3, MMP-7, MMP-9, TIMPs	Macrophages and inflammatory factors activate MMPs, leading to degradation of extracellular matrix components in the NP and AF, including collagen and proteoglycans.	(200, 201)
	Autophagy and apoptosis	MAPK signaling pathway, PI3K/AKT pathway, autophagy markers	Inflammatory factors induce apoptosis in NP cells, directly reducing the volume of the herniated tissue. Meanwhile, autophagy is activated, which can protect cells but may also mediate cell death when excessively activated, thereby jointly promoting tissue degradation.	(202, 203)
Physical and biochemical factors	IVD dehydration	Aggregated proteoglycans	After the herniated tissue is exposed to the epidural environment, the highly osmotic NP tissue becomes dehydrated, leading to volume reduction.	(199, 204)
	Mechanical tension	Posterior longitudinal ligament	The posterior longitudinal ligament exerts mechanical traction on the herniated NP tissue, which may cause “mechanical retraction,” promoting NP retraction or altering its morphology.	(205)

AF, annulus fibrosus; ECM, extracellular matrix; IVD, intervertebral disc; NP, nucleus pulposus; VEGF, vascular endothelial growth factor; bFGF, basic fibroblast growth factor; VEGF-D, vascular endothelial growth factor-D; VEGFR-3, vascular endothelial growth factor receptor-3; MMP, matrix metalloproteinase; TIMP, tissue inhibitor of metalloproteinase; MAPK, mitogen-activated protein kinase; PI3K/AKT, phosphoinositide 3-kinase/protein kinase B.

In addition, lymphatic vessels have also been shown to participate in the above resorption process. Fu et al. (194) found that lymphangiogenesis was observed in herniated NP tissue from patients with LDH. Meanwhile, in a mouse model of NP migration into paravertebral tissue (NPH), NP cells were detected in the paravertebral tissue and the draining lymph nodes (dLNs) at 4 hours; 24 hours later, NP cells in the dLNs increased significantly; after approximately 1 week, most NP cells in the paravertebral tissue had been cleared, whereas IVD inflammation peaked at 1 week and lymphangiogenesis persisted. More importantly, conditional VEGFR3 knockout mice exhibited impaired lymphangiogenesis and impaired NP cell clearance, accompanied by aggravated IVD inflammation.

The role of vascular and lymphatic system remodeling in herniated tissue resorption can be summarized in two aspects. First, it serves as a transport route, delivering more peripheral immune cells to the lesion site to amplify the clearance effect, while draining inflammation-mediated degradation products to prevent excessive amplification and persistence of local inflammation. Second, it serves as a functional support platform, supporting immune cell activation and the synthesis of matrix-degrading enzymes by providing nutritional supply and a suitable microenvironment, thereby indirectly promoting the clearance of herniated tissue.

### 5.3 Alterations in the immune microenvironmental landscape

Remodeling of the vascular and lymphatic systems can markedly alter the original immune microenvironment of the IVD, as manifested by increased immune cell infiltration and aggravated local inflammatory responses, thereby affecting matrix degradation and accelerating the degenerative process (127). The formation of neovessels directly disrupts the physical barrier of the IVD and its avascular nature, thereby breaking the tissue’s inherent immune-privileged state (14–17). Immune effector components in the circulation, including immune cells, antibodies, and complement, can enter IVD tissue through neovessels, while self-antigens in the NP that have long been isolated from the immune system, such as proteoglycan fragments and collagen, are thereby exposed to immune surveillance, potentially creating conditions for the development of autoimmune responses (44, 105, 127).

In addition to barrier disruption, remodeling of the vascular and lymphatic systems also affects the degenerative process by regulating related molecules. In degenerative IVDs, although increased VEGF expression may facilitate nutrient supply within the IVD, the expression of pro-inflammatory factors such as MMP-3 is also upregulated, inhibiting the synthesis of type II collagen and thereby accelerating the degenerative process of the IVD (29). In addition, the reduced number of lymphatic vessels after IVD degeneration is associated with impaired clearance of immune cells and metabolic waste products, as well as the persistence of local inflammatory responses (171). This association suggests that lymphatic functional decline contributes to alterations in the degenerative microenvironment, but this still requires validation through functional lymphatic drainage experiments.

### 5.4 Clinical implications and stratified interventions: from biphasic effects to precision assessment

Remodeling of the vascular and lymphatic systems exerts divergent effects across different pathological processes of IDD. Therefore, the choice between strategies to “inhibit vasculogenesis” and to “promote vasculogenesis” depends on noninvasive assessment of the vascular and lymphatic status of the IVD. The characteristic enhancement patterns observed on contrast-enhanced MRI can be used to indicate vascularization of the herniated tissue and to assist in predicting the resorption trend (206). Dynamic contrast-enhanced MRI (DCE-MRI) can quantify blood flow, blood volume, and vascular permeability (207), while intravoxel incoherent motion diffusion-weighted imaging (IVIM-DWI) can provide parameters reflecting the true diffusion of water molecules and microcirculatory perfusion (208). These techniques are expected to distinguish the “IVD inflammatory phase” from the “IVD repair phase” and to be used for therapeutic monitoring and progression prediction. In addition, imaging features such as endplate vascular channels and calcification may coexist with local inflammation–vascular remodeling activity and may serve as auxiliary clues for identifying “high-activity degeneration.” Such a subtype may be preliminarily defined as showing a high degree of endplate vascularization on imaging, with or without subchondral bone marrow edema, and may biologically correspond to a state characterized by a heavier local inflammatory burden and active vascular and lymphatic remodeling (47, 209, 210).

Hematological markers can provide complementary information beyond imaging assessment. Plasma tenascin-C and circulating miR-155-5p levels correlate closely with herniation volume and help evaluate the remodeling status and spontaneous resorption tendency of herniated tissue (211). Serum SDF-1/CXCL12 participates in multiple processes including inflammatory cell chemotaxis, vascular endothelial migration, and nerve axon guidance; changes in its levels may reflect activation states of pathways associated with inflammation, angiogenesis, lymphangiogenesis, and nerve ingrowth (124). Methylmalonic acid (MMA), a senescence-associated metabolite significantly elevated in degenerative IVDs, promotes vascularization via the CCL7/JAK2-STAT3/VEGF pathway and may aid in identifying IVD neovascularization driven by senescence-associated metabolic dysregulation (212).

By integrating imaging and hematological findings, clinicians can preliminarily determine the nature, pathological significance, and prognostic trajectory of vascular and lymphatic remodeling, providing a basis for distinguishing the inflammatory phase from the repair phase of IVD disease, monitoring treatment response, determining intervention direction, and optimizing follow-up strategies. However, specificity and threshold criteria for the aforementioned markers still require further validation and standardization in multicenter prospective studies with clearly defined grading systems and sufficient sample sizes.

## 6 Therapeutic strategies targeting the vascular and lymphatic systems and future perspectives

Given the critical role of vascular and lymphatic system remodeling in the progression of IDD, targeting the vascular and lymphatic systems has emerged as an important therapeutic strategy for IDD. On the basis of the above-mentioned precision assessment, stratified decision-making according to clinical manifestations, anatomical location, and key molecular mechanisms, followed by tailored interventions, may help effectively alleviate or even reverse IDD progression.

### 6.1 Inhibition of pathological vascularization: intervention centered on the pain-dominant stage

In contexts where discogenic pain is the predominant clinical phenotype, pathological angiogenesis is often coupled with the ingrowth of nerve fibers and inflammatory cell infiltration; thus, inhibition of vascularization is regarded as a translatable strategy with clear pathological targeting specificity (36, 37, 183). Animal studies suggest that local anti-angiogenic interventions can reduce vascular and neural ingrowth and improve pain-related behavioral outcomes (39, 188).

Drawing on anti-angiogenic therapeutic strategies used in tumors, Chen et al. (213) applied a bevacizumab-loaded thermosensitive hydrogel to a rat model of IDD and found that it effectively inhibited VEGF expression, thereby delaying IDD. Traditional Chinese medicine therapies have also shown potential in regulating angiogenesis. Zhang et al. (85) found that moxibustion could enhance autophagy and reduce apoptosis via the HIF-1α/VEGF pathway by inhibiting VEGF expression in NP cells, thereby delaying the progression of IDD. Physical therapy represents another important direction for intervention. Hwang et al. (214) found that photobiomodulation (PBM), as a noninvasive physical irradiation therapy, significantly inhibited VEGF production and expression, blocked endothelial cell proliferation, downregulated the expression of inflammatory factors and MMPs, and attenuated the ability of microvessels to penetrate from the AF into the NP; this effect was regulated through the MAPK signaling pathway. Electrical stimulation (ES) has also been shown to reduce the expression of inflammatory factors and MMPs within the IVD. Using a microfluidic platform, Jin et al. (215) found that electrical stimulation significantly inhibited macrophage-mediated inflammatory responses, thereby reducing inflammation-associated microangiogenesis.

In addition to directly targeting VEGF, strategies aimed at upstream inflammatory drivers or synergistic nodes also hold potential. Based on the synergistic role of the “IL-1β–VEGF–NGF” axis in inflammation, vascularization, and nerve ingrowth (48, 123), antagonizing IL-1β, blocking NGF signaling, or inhibiting the VEGF/VEGFR axis may all serve as potential intervention approaches. In addition, inhibiting signaling components associated with endothelial migration and protease expression, such as Rac1/PAK1, or reducing immune cell-derived pro-angiogenic stimuli by stabilizing mast cells and blocking PAR2 signaling (115, 216, 217), also requires further validation of feasibility in future studies.

### 6.2 Improving endplate microcirculation: nutrition-oriented intervention strategies

One of the important upstream events in IDD is impaired nutrient diffusion caused by CEP calcification and restricted microcirculation (47). Improving the structure and function of microvessels in the endplate region represents a new entry point for delaying degeneration. Vascular endothelial cells play a critical role in maintaining microvascular structure and function. Using an aged mouse model, Chen et al. (82) found that vascular endothelial cell senescence was positively correlated with vascular degeneration and a reduction in the number of microvessels within the bony endplate, leading to a marked decrease in nutrient supply to the IVD; conversely, senolytic drugs effectively cleared senescent endothelial cells from the bone marrow cavity of the bony endplate, stimulated endothelial cell neogenesis, and increased microvascular density by upregulating VEGF expression, thereby delaying IDD progression. As a calcium channel blocker, nimodipine can effectively reduce vascular tone and increase blood flow (218). Gulbrandsen et al. (219) found that nimodipine significantly increased microvascular length within the bony endplate and the contact length between these microvessels and the cartilage endplate, thereby promoting nutrient transport across the endplate–nucleus pulposus interface and delaying IDD degeneration. Notably, although nicotine can increase vascular number and area, it does not enhance material transport or nutrient supply capacity, suggesting that an increase in vascular quantity is not equivalent to functional improvement.

### 6.3 Enhancing endogenous repair: combination strategies supported by cell-based and delivery technologies

Beyond “inhibiting harmful processes” and “improving endplate microcirculation,” strategies aimed at enhancing endogenous repair and reconstructing a homeostatic microenvironment may represent potential therapeutic approaches. Their core lies in using cells as carriers or materials as platforms to achieve coordinated regulation of inflammation, matrix remodeling, and vascular and lymphatic remodeling. NCs and their paracrine products can suppress the angiogenic phenotype of endothelial cells under hypoxic conditions and influence angiogenesis through signaling delivered by soluble factors or exosomes (31). Approaches such as NC transplantation, NC-conditioned medium, or exosome-based delivery may help reconstruct a local “low-inflammation, anti-vascular/anti-neural ingrowth” microenvironment within the degenerated IVD (133, 220). The key issues in this direction lie in the source of the cells and the stability of their phenotype, the delivery vehicle and retention capacity, and stratified validation of the net effects across different stages of degeneration.

Engineered immune cell strategies, by contrast, target the sustained amplification of inflammation caused by insufficient clearance of apoptotic cells and cellular debris in degenerative tissues. By enhancing the phagocytic and clearance capacities of macrophages, such as through upregulation of phagocytic receptors, these strategies improve clearance and repair efficiency, thereby reducing the inflammatory drive associated with the persistent release of danger-associated molecules and promoting the secretion of anti-inflammatory and tissue repair factors (221).

In addition, the role of the lymphatic system in IDD also warrants attention. As an important route for immune cell trafficking and inflammatory mediator drainage, improvement of lymphatic vessel function may enhance local inflammatory clearance capacity and complement vascular regulation (19, 171). Existing studies suggest that enhanced lymphangiogenesis is associated with inflammatory cell clearance and preservation of tissue structure (171), thereby providing a new interventional perspective for regulating the “immune–lymphatic–vascular” network.

### 6.4 Delivery technologies and translational bottlenecks

The low vascularity and ECM of the IVD determine that drug penetration is strongly dependent on molecular size, charge, protein-binding rate, and retention capacity; thus, achieving efficient and sustained local delivery remains a major translational bottleneck (222, 223). Excessive or nonselective inhibition of angiogenesis may impair the microcirculation and nutrient diffusion necessary in the endplate region and may instead aggravate metabolic dysfunction (82). Therefore, the feasibility of interventional strategies largely depends on the spatial specificity of administration, controllability of release, and selection of the therapeutic window, with priority given to targeting pathological intradiscal vascularization rather than the nutritional blood supply of the endplate.

Delivery platforms represented by nanocarriers and physical field-assisted transfection can improve the construction efficiency and controllability of therapeutic nucleic acids or engineered cells (224–226). Local delivery systems, such as dissolvable microneedle arrays, injectable hydrogels, and nanoparticles, can achieve precise localization and sustained release of drugs and biologics with less structural disturbance (227–229).

**Figure 5:**
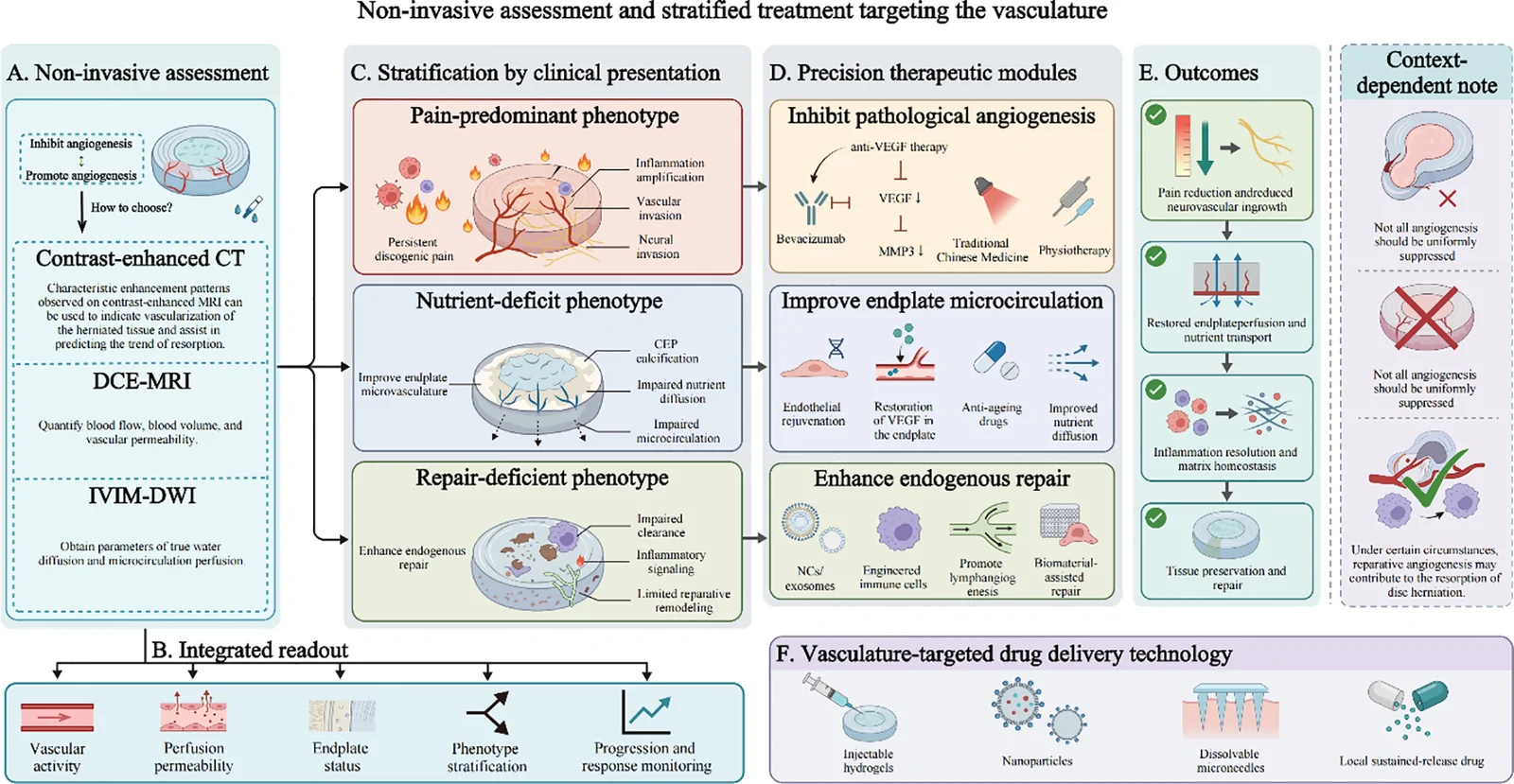
Non-invasive assessment and stratified treatment targeting the vasculature. **(A)** Non-invasive imaging modalities for assessing vascular alterations in intervertebral disc degeneration. Contrast-enhanced CT can identify characteristic enhancement patterns associated with neovascularization, herniated tissue, and potential resorption tendency; dynamic contrast-enhanced MRI (DCE-MRI) enables quantitative assessment of blood flow, blood volume, and vascular permeability; and intravoxel incoherent motion diffusion-weighted imaging (IVIM-DWI) provides parameters reflecting water diffusion and microcirculatory perfusion. **(B)** Integrated imaging readouts include vascular activity, perfusion permeability, endplate status, phenotype stratification, and longitudinal monitoring of disease progression and therapeutic response. **(C)** Imaging and clinical features may support stratification into distinct vasculature-associated phenotypes, including a pain-predominant phenotype characterized by persistent discogenic pain, inflammatory amplification, vascular invasion, and neural ingrowth; a nutrient-deficit phenotype associated with impaired endplate microcirculation, cartilaginous endplate calcification, impaired nutrient diffusion, and reduced microvascular supply; and a repair-deficient phenotype characterized by impaired clearance, persistent inflammatory signaling, and limited reparative remodeling. **(D)** Precision therapeutic modules include inhibition of pathological angiogenesis, improvement of endplate microcirculation, and enhancement of endogenous repair. Anti-angiogenic strategies may target VEGF-related pathways and downstream mediators, while endplate microcirculation may be improved through endothelial rejuvenation, modulation of VEGF signaling in the endplate, anti-ageing interventions, and promotion of nutrient diffusion. Endogenous repair may be enhanced by notochordal cell-derived exosomes, engineered immune cells, lymphangiogenesis-promoting approaches, and biomaterial-assisted repair. **(E)** Expected therapeutic outcomes include pain reduction with decreased neurovascular ingrowth, restoration of endplate perfusion and nutrient transport, inflammation resolution with recovery of matrix homeostasis, and preservation or repair of disc tissue. The context-dependent note highlights that angiogenesis should not be uniformly suppressed, as reparative angiogenesis may, under certain conditions, contribute to disc herniation resorption. **(F)** Vasculature-targeted drug delivery platforms, including injectable hydrogels, nanoparticles, dissolvable microneedles, and local sustained-release systems, may enable spatially controlled and phenotype-adapted therapeutic intervention.

Overall, therapeutic strategies targeting the vasculature should shift from a singular focus on “promoting or inhibiting angiogenesis” to an integrated approach based on disease-stage stratification, regional differences, and multi-target synergy. During stages dominated by inflammation and pain, priority should be given to suppressing pathological neurovascularization within the disc and promoting local lymphangiogenesis to enhance inflammatory clearance; during the stage of endplate nutritional impairment, however, restoration of nutrient supply should be achieved by improving microcirculation. Combination strategies integrating engineered cells with intelligent delivery technologies may help overcome the current bottlenecks in IVD-targeted intervention (**Figure 5**).

## 7 Conclusions

During the progression of IDD, pathological angiogenesis occurs in the originally avascular NP and inner AF. This change is not an isolated histological phenomenon but arises from the coupled effects of multiple factors, including disruption of anatomical barriers and imbalance of matrix homeostasis (36, 37), upregulation of pro-angiogenic signals (38), downregulation of anti-angiogenic signals (39), and inflammatory responses (40). On the one hand, blood vessels often grow alongside nerve fibers, forming “neurovascular complexes,” which provide a structural basis for discogenic pain (36, 37, 183). On the other hand, angiogenesis also plays a beneficial role in the resorption of IVD herniations by promoting immune cell recruitment and tissue clearance, thereby participating in disease resolution (192). Compared with blood vessels, the lymphatic system in the IVD has been less extensively studied. Existing evidence suggests that, under physiological conditions, lymphatic structures are mostly confined to the peridiscal connective tissue and the outer AF (171, 173, 175), and that changes in lymphatic structure and function during degeneration may affect the efficiency of inflammatory clearance and the local immune microenvironment (171). However, whether lymphatic structures in the IVD form lymphatic vessels with drainage function remains to be verified through more direct tissue-clearing techniques and drainage-tracing experiments. In addition, targeting the vascular and lymphatic systems represents a therapeutic strategy with translational potential; the key lies in establishing a more precise noninvasive assessment system for the IVD and, supported by engineered cells and intelligent delivery, promoting the translation of basic research into clinically stratified interventions.
